# Uncovering a role for METTL13 in malignant transformation of human hematopoietic stem cells and in the progression of pediatric leukemia

**DOI:** 10.1038/s41419-026-08761-7

**Published:** 2026-04-25

**Authors:** Sabina Enlund, Chae-Eun Lim, Isabella Hoang, Sonali Joshi, Maria Rivera, Adena Pepich, Amanda Ramilo Amor, Jacob Short, Cecilia Thomsson, Indranil Sinha, Shahrzad Shirazi Fard, Anna Nilsson, Ola Hermanson, Qingfei Jiang, Frida Holm

**Affiliations:** 1 Deparment of Women’s and Children’s Health, Division of Pediatric Oncology and Surgery, Karolinska Institutet, 17177 Stockholm, Sweden; 2 Division of Regenerative Medicine, Department of Medicine, Moores Cancer Center, University of California, San Diego, La Jolla, CA 92037 USA; 3 Department of Neuroscience, Karolinska Institutet, 17177 Stockholm, Sweden; 4 Department of Laboratory Medicine, Karolinska Institutet, 14152 Huddinge, Sweden

**Keywords:** Cancer stem cells, Acute lymphocytic leukaemia

## Abstract

Post-transcriptional RNA modifications, such as N6-methyladenosine (m6A) methylation and adenosine to inosine (A-to-I) editing, are critical regulators of hematopoietic stem cell (HSC) self-renewal and differentiation, yet their precise contributions to malignant transformation are not fully elucidated. In this study, we uncovered the epitranscriptomic landscape caused by knockdown of genes from the methyltransferase (METTL)-family in hematopoietic stem and progenitor cells (HSPCs). We identified both converging and distinct effects of METTL3 and METTL14, known members of the m6A writer complex, as well as orphan gene METTL13. Amongst METTL-family members, only METTL13 transcription was increased following adenosine deaminase acting on RNA 1 (ADAR1) overexpression in HSPCs. This transcriptional pattern suggests that METTL13 may participate in biological programs that partially overlap with those controlled by the m6A writer complex and ADAR1, although any mechanistic relationship remains undefined. Knockdown of METTL13 altered the expression of multiple genes involved in oncogenic development in HSPCs. Furthermore, METTL13 expression was associated with a high-risk profile in pediatric T-cell acute lymphoblastic leukemia (T-ALL) and functional studies confirmed that METTL13 is required for T-ALL cell proliferation and survival both in vitro and in vivo. Collectively, our results identify METTL13 as a previously unrecognized regulator of leukemic transformation, independent of any presumed mechanistic interaction between RNA editing and m6A pathways.

## Introduction

Epitranscriptomic alterations, which comprise numerous post-transcriptional RNA modifications, have emerged as important drivers of disease development and cancer progression [1]. RNA modifications shape the cellular response to environmental cues, by regulating cell survival, differentiation and migration. Aberrant activity of these RNA modifications can contribute to malignant transformation and has been linked to a wide variety of cancers [2].

Among RNA modifications, N6-methyladenosine (m6A) methylation and adenosine to inosine (A-to-I) editing are the most abundant, both targeting adenosines on mRNA to alter its fate and function [3]. A-to-I editing is catalyzed by the adenosine deaminase acting on RNA (ADAR)-family, primarily ADAR1 and ADAR2, although ADAR1 is the predominant enzyme in regulating innate immunity and maintaining hematopoietic stem cells (HSCs) [4–6**]**. Aberrant A-to-I RNA editing impairs stem cell fitness and promotes cancer development [7–9**]**. Malignant overexpression of ADAR1 has been reported in over 20 types of cancers, including pediatric T-cell acute lymphoblastic leukemia (T-ALL) [7, 10].

RNA methylation by the m6A complex is a dynamic process mediated by a large network of proteins, including the core writer complex (methyltransferases (METTLs) METTL3 and METTL14), erasers (fat mass and obesity-associated (FTO)) and demethylase AlkB homolog 5 (ALKBH5) and several readers [3, 11–17]. Modifications by the m6A complex affect many aspects of mRNA metabolism, including mRNA stability, splicing, and translation [18**–**21]. Many of m6A functions overlap with those of ADAR1. Both highly regulated m6A methylation and A-to-I editing are required for HSC self-renewal and differentiation [4–6, 8, 22–27], and malignant activity of these modifications can promote the development of both solid tumors and hematological cancers^,^ [27**–**31]. Although both m6A and A-to-I RNA modifications target adenosine bases, A-to-I RNA editing occurs mainly in inverted Alu sequences enriched in intronic and 3’UTR regions, while m6A are located within highly conserved RRACH sequences within 3’UTR and exons [25, 32]. Whether these two RNA-modifying processes functionally interact to regulate HSC fitness and promote malignant transformation to leukemia is yet to be defined.

Although the role of the m6A complex has been extensively studied in the context of cancer progression, there are numerous members of the METTL-family whose role in malignant development have not been elucidated. Recent reports have identified METTL13 as a lysine methyltransferase that modifies the eEF1a protein at lysine 55, thus promoting translation [33, 34]. METTL13 has been correlated with a decreased survival probability in malignancies such as lung and pancreatic cancer and to augment metastasis in gastric cancer [34, 35]. Existing studies focus mainly on methylation of eEF1A and changes in translational output, several highlighting the potential oncogenic role of METTL13 in cancer. In contrast, loss of METTL13 was considered a poor prognostic marker in clear cell renal cell carcinoma, inhibiting proliferation and metastatic capacity of cancer cells [36]. Emerging research has identified METTL13 as regulator of proliferation and survival in acute myeloid leukemia (AML), where high levels of METTL13 correlated with a poor prognosis [37]. Despite this, the role of METTL13 in hematological malignances and HSC biology remains largely unclear.

In this study, we investigated the role of METTL-family genes in HSC maintenance and hematological malignancies. Since ADAR1 overexpression alters global transcriptional programs in HSPCs, including genes involved in RNA modification, we examined whether METTL-family expression patterns change in this context, and potentially converge with ADAR1 in transcriptional regulation. We report that overexpression of ADAR1 in hematopoietic stem and progenitor cells (HSPCs) suppressed most m6A-related genes, except for METTL13, an orphan METTL-family member whose function in HSCs and leukemia stem cells (LSCs) has not been reported. Knockdown of METTL13 disrupted many immune signaling and survival pathways convergent with METTL3 and METTL14, while uniquely affecting various cancer-related pathways. We also investigated the role of METTL13 in pediatric acute lymphoblastic leukemia (ALL) and found that increased METTL13 expression correlated with a poor prognosis in both T-ALL and B-cell ALL (B-ALL). Loss of METTL13 in T-ALL cells led to arrested cell proliferation and reduced cell viability, both in vivo and in vitro, and suppressed pathways regulating proliferation and DNA-replication. Collectively, our findings reveal consequences of alterations to the METTL-family in disease development and recognizes an emerging role for METTL13 in hematological malignancies.

## Results

### ADAR1 overexpression downregulates members of the m^6^A complex in HSPCs

**Fig. 1:**
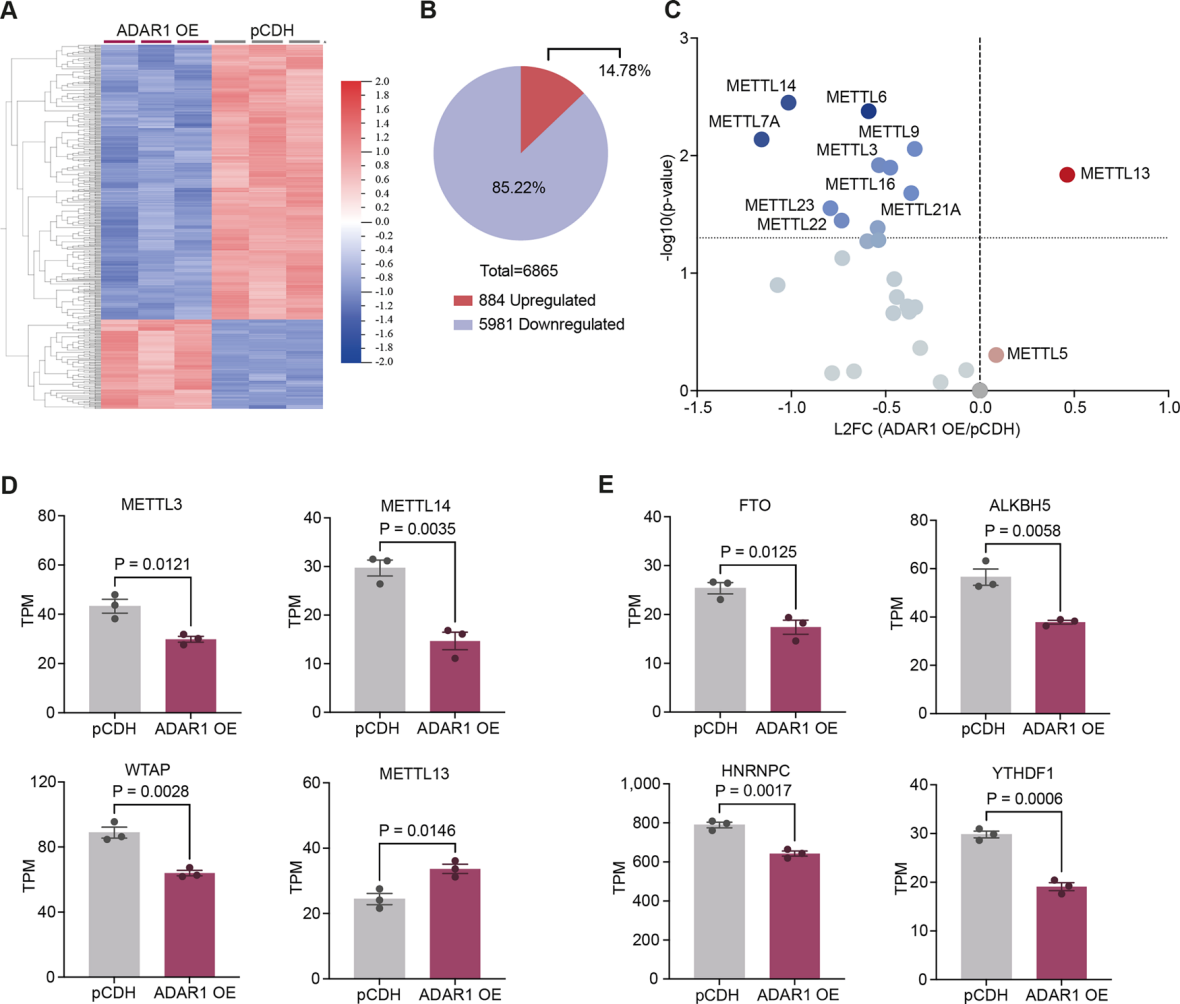
ADAR1 overexpression suppressed expression of members from the m6A complex. **A** Heat map of the top 500 differentially expressed genes in ADAR1 overexpressed human CD34^+^ HSPCs (ADAR1 OE, *n* = 3) compared to the backbone control (pCDH, *n* = 3). Created in Qlucore Omics Explorer, significance was calculated using unpaired two-tailed *t*-test, *p* < 0.05. **B** Distribution of differentially expressed genes in ADAR1 overexpressed cells (*n* = 3) compared to the pCDH control (*n* = 3). Significance was calculated using unpaired two-tailed t-test, *p* < 0.05. **C** Volcano plot of dysregulated genes from the METTL-family following ADAR1 overexpression (*n* = 3 per condition). Significance was calculated using unpaired two-tailed t-test, results are displayed as L2FC (ADAR OE/pCDH) and negative log10 *p*-value. **D** Expression of genes from the m6A writer complex (METTL3, METTL14 and WTAP) and from the METTL-family (METTL13) in ADAR1 overexpressed cells (*n* = 3) compared to compared to the pCDH control (*n* = 3). Significance was calculated using unpaired two-tailed t-test, results are displayed as TPM, mean ± SEM. **E** Significantly differentially expressed m6A erasers FTO and ALKBH5 and readers HNRNPC and YTHDF1 in ADAR1 overexpressed cells (*n* = 3) compared to compared to the pCDH control (*n* = 3). Significance was calculated using unpaired two-tailed t-test, results are displayed as TPM, mean ± SEM.

To investigate if ADAR1 transcriptionally regulates members of the m6A complex, we examined effects following lentiviral overexpression of ADAR1 (p150 isoform) in human CD34^+^ HSPCs (available at BioProject: PRJNA319866) (Fig. SF1A). A total of 6865 genes were significantly differentially expressed in ADAR1 overexpressed HSPCs compared to the backbone control (pCDH), with the majority (> 85%) of genes being downregulated (Fig. 1A, B, Supplemental Table 1). By focusing on the m6A regulatory gene network, we found that overexpression of ADAR1 led to a downregulation of the main m6A writer complex (METTL3, METTL14 and WTAP) [38**–**40], m6A erasers (FTO and ALKBH5), and readers (HNRNPC and YTHDF1) (Fig. 1D, E). Other suppressed methyltransferase-like genes included METTL7A, a thiol methyltransferase [41]; METTL9, a methyltransferase for 1-methylhistidine [42]; and METTL16, which functions as both a non-conical nuclear m6A writer and cytosolic translation-initiator [43] (Fig. SF1B). The only significantly upregulated gene from the METTL-family was METTL13. Interestingly, overexpression of an editing defective ADAR1 mutant (ADAR1^E912A^) in HSPCs, displayed similar suppressive effects on many members from the METTL-family and the m6A complex, while still upregulating METTL13 (Fig SF1C, Supplemental Table 1). Together, these results indicate a potential transcriptionally suppressive relationship between ADAR1 and most METTL-family members, except for METTL13, likely through RNA editing-independent capacities of ADAR1.

### Overlapping and distinct gene regulation programs by METTL3, METTL9, METTL13 and METTL14 in HSPCs

**Fig. 2:**
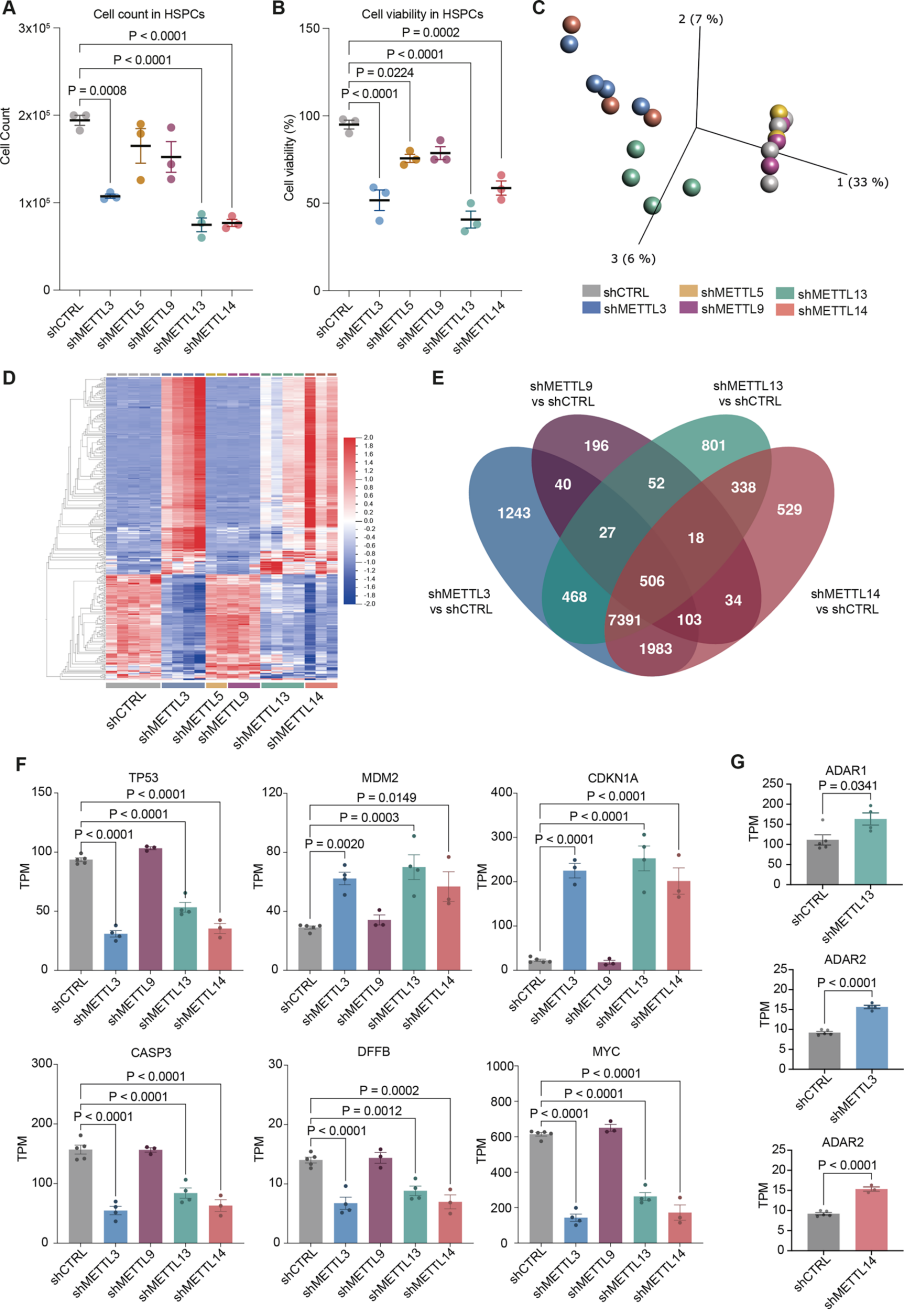
Overlapping and distinct gene regulation by METTL3, METTL9, METTL13 and METTL14 in HSPCs. **A** Total cell counts following METTL knockdown (shMETTL3 (*n* = 3), sMETTL5 (*n* = 3), shMETTL9 (*n* = 3), shMETTL13 (*n* = 3) and shMETTL14 (*n* = 3)) in HSPCs compared to the control (shCTRL, *n* = 3) in HSPCs. Significance was calculated by ordinary one-way ANOVA with multiple comparisons compared to shCTRL, as well as Dunnett correction. **B** Cell viability in percentage following METTL knockdown (shMETTL3 (*n* = 3), sMETTL5 (*n* = 3), shMETTL9 (*n* = 3), shMETTL13 (*n* = 3) and shMETTL14 (*n* = 3)) in HSPCs compared to the control (shCTRL, *n* = 3) in HSPCs. Significance was calculated by ordinary one-way ANOVA with multiple comparisons compared to shCTRL, as well as Dunnett correction. **C** PCA plot of METTL3 (shMETTL3, *n* = 4), METTL5 (shMETTL5, *n* = 2), METTL9 (shMETTL9, *n* = 3), METTL13 (shMETTL13, *n* = 4) and METTL14 (shMETTL14, *n* = 3) knockdown in human CD34^+^ HSPCs compared to the backbone control (shCTRL, *n* = 5). Created in Qlucore Omics Explorer. **D** Heatmap of shMETTL3 (*n* = 4), shMETTL5 (*n* = 2), shMETTL9 (*n* = 3), shMETTL13 (n = 4) and shMETTL14 (*n* = 3) in HSPCs compared to shCTRL (*n* = 5). Created in Qlucore Omics Explorer, significance was calculated using multi-group ANOVA, *q* < 0.1, SD < 0.05. **E** Venn diagram of differentially expressed genes in shMETTL3 (*n* = 4), shMETTL5 (*n* = 2), shMETTL9 (*n* = 3), shMETTL13 (*n* = 4) and shMETTL14 (*n* = 3) in HSPCs compared to shCTRL (*n* = 5). Significance was calculated using unpaired two-tailed t-test, *p* < 0.05, in each shMETTL compared to shCTRL. **F** Dysregulated genes (TP53, MDM2, CDKN1A, CASP3, DFFB and c-MYC) in shMETTL3 (*n* = 4), shMETTL5 (*n* = 2), shMETTL9 (*n* = 3), shMETTL13 (*n* = 4) and shMETTL14 (*n* = 3) in HSPCs compared to shCTRL (*n* = 5). Significance was calculated using ordinary one-way ANOVA with multiple comparisons compared to shCTRL, as well as Dunnett correction, results are displayed as TPM, mean ± SEM. **G** Differentially expressed genes from the ADAR family in shMETTL3 (*n* = 4), shMETTL13 (*n* = 4 and shMETTL14 (*n* = 3) compared to shCTRL (*n* = 5). Significance was calculated using unpaired two-tailed t-test, results are displayed as TPM, mean ± SEM.

We systematically studied the gene expression landscape of several m6A family members affected by ADAR1 in human CD34^+^ HSPCs. Although the role of METTL3 and METTL14 are well established in HSC maintenance, METTL5, 9, and 13 have not been extensively studied in this context. To achieve this, lentiviral knockdown of METTL3, METTL5, METTL9, METTL13 and METTL14 was performed individually in CD34^+^ HSPCs, followed by whole transcriptome RNA-sequencing (Fig. SF2A, SF2B). The protein level knockdown of METTL13 was confirmed by western blot (SF2C, SF2D, uncropped western blots Fig. A). Knockdown of METTL3, METTL13 and METTL14 significantly reduced total cell number and cell viability in HSPCs, while knockdown of METTL5 also impaired cell viability, to a lesser extent (Fig. 2A, B). Principal component analysis (PCA) revealed three potential outlier samples, which were excluded in the subsequent analysis (Fig. SF2E). METTL3 and METTL14 knockdown conditions clustered together, separated from the control (Fig. 2A). Both shMETTL5 and shMETTL9 grouped together with the control, suggesting that METTL5 and METTL9 may have less effect on a transcriptional level, although knockdown efficacy requires improvement for further conclusions (Fig. 2C, D). Knockdown of METTL13 had similar effects on gene expression as METTL3 and METTL14, yet shMETTL13 HSPCs formed their own cluster away from the other METTLs in PCA, suggesting both overlapping and unique functions (Fig. 2C, D).

Next, we analyzed differentially expressed genes in each individual METTL knockdown condition compared to the control (Fig. 2E, SF2F, Supplemental Table 2). We excluded METTL5 due to low power after RNA quality control. Knockdown of METTL3, METTL13 and METTL14 shared 7391 differentially expressed genes, while knockdown of METTL3 and METTL14 alone only shared 1983 genes (Fig. 2E). Our results suggest that the gene expression program of METTL13 substantially overlaps with that of m6A writer complex. Out of the overlapping genes, reduced expression of tumor suppressor TP53 and upregulation of its negative regulator MDM2 were reported (Fig. 2F) [44]. Moreover, CDKN1A, an effector protein downstream of p53, was upregulated [45]. Knockdown also suppressed apoptotic genes, including CASP3 and DFFB [46], and oncogenes such as c-MYC [47].

In the light of reported links between m6A pathways and ADAR1 biology [48], we examined ADAR1 transcript levels after METTL knockdown in HSPCs. We observed transcriptional upregulation of ADAR1 following METTL13 knockdown (Fig. 2G). METTL3 and METTL14 knockdown caused an upregulation of ADAR2, which is mainly expressed and active in brain tissue, rather than the hematopoietic system (Fig. 2G) [7]. This data indicates a potential suppressive relationship between METTL-family members and ADAR1 but would need further validation to confirm if ADAR1 protein levels or activity are altered. Consistent with context-dependent regulation, ADAR1 protein abundance was unchanged upon METTL13 knockdown in T-ALL cell lines (Fig SF2G–H, uncropped western blots Fig. B). Thus METTL13-loss phenotype in T-ALL is compatible with ADAR1-abundance-independence mechanism. Taken together, these data suggest that METTL13 converges with METTL3 and METTL14 in regulating gene expression and cell viability in HSPCs, while its distinct PCA clustering and unique gene expression profile indicates that METTL13 may have some unique properties.

### METTL3, METTL13 and METTL14 converged in regulating immune signaling while METTL13 caused distinct effects on apoptosis and p53 regulation

**Fig. 3:**
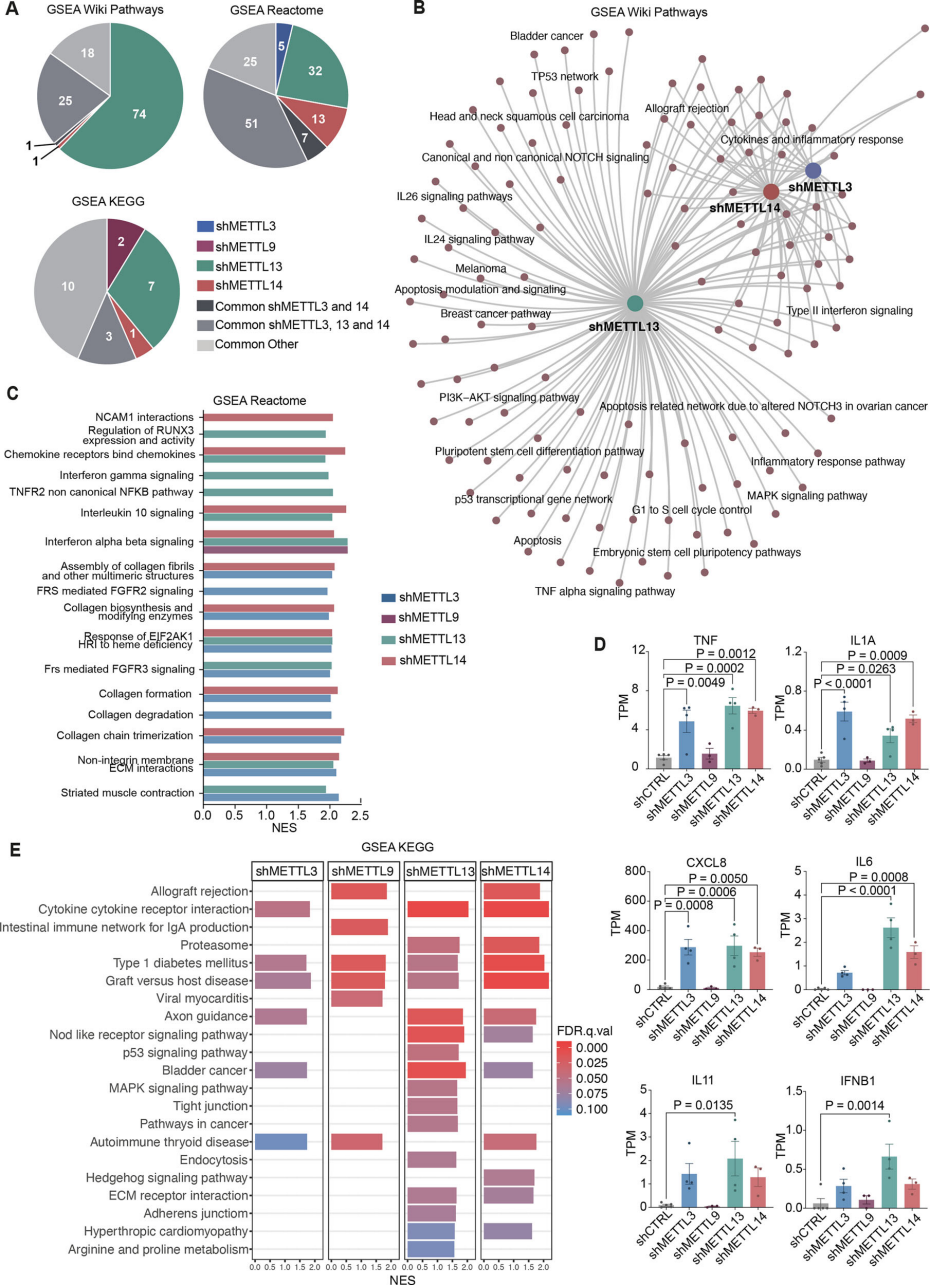
METTL3, METTL13 and METTL14 converged in regulating immune signaling, with many distinct pathways altered uniquely by METTL13. **A** Distribution of significantly enriched pathways in each shMETTL compared to shCTRL generated through GSEA. Created in GSEA and MSigDB, FDR *q* < 0.1, including only protein-coding genes, using three different gene set libraries (Wiki Pathways, Reactome and KEGG). Groups are based on which pathways are uniquely altered by shMETTL3 (*n* = 4), shMETTL9 (*n* = 3), shMETTL13 (*n* = 4) or shMETTL14 (*n* = 3), as well pathways altered in several conditions; the m6A writer complex (METTL3 and METTL14) with and without METTL13, as well as all other combined conditions, compared to shCTRL (*n* = 5) in human CD34^+^ HSPCs. **B** Network plot of GSEA Wiki Pathways in shMETTL3 (*n* = 4), shMETTL13 (*n* = 4 and shMETTL14 (*n* = 3) compared to shCTRL (*n* = 5) (FDR *q* < 0.1). **C** Top 10 significant GSEA Reactome pathways in shMETTL3 (*n* = 4), shMETTL9 (*n* = 3), shMETTL13 (*n* = 4) and shMETTL14 (*n* = 3) compared to shCTRL (*n* = 5) (FDR *q* < 0.1). Results are displayed as normalized enrichment score (NES). **D** Dysregulated genes involved in inflammatory signaling (TNF, IL1A, CXCL8, IL6, IL11 and IFNB1) in shMETTL3 (*n* = 4), shMETTL9 (*n* = 3), shMETTL13 (*n* = 4) and shMETTL14 (*n* = 3) compared to shCTRL (*n* = 5). Significance was calculated using ordinary one-way ANOVA with multiple comparisons compared to shCTRL, as well as Dunnett correction, results are displayed as TPM, mean ± SEM. E) GSEA of shMETTL3 (*n* = 4), shMETTL9 (*n* = 3), shMETTL13 (*n* = 4) and shMETTL14 (*n* = 3) compared to shCTRL (*n* = 5) using KEGG legacy pathways (FDR *q* < 0.1) Results are displayed as NES and FDR q-value.

To uncover signaling pathways regulated by METTL3, METTL9, METTL13 and METTL14 in HSPCs, we performed Gene set enrichment analysis (GSEA) on each shMETTL compared to shCTRL, utilizing the WIKI Pathway, Reactome and KEGG libraries. Notably, METTL13 knockdown resulted in the highest number of uniquely altered pathways across all three libraries (Figs. 3A, SF3A, SF3B, SF3C). By contrast, majority of the pathways that were altered by the core writer complex (METTL3 and METTL14) overlaps with METTL13, while METTL3 and METTL14 knockdown alone caused minimal overlap.

METTL13 knockdown exclusively enriched 74 Wiki Pathways, with 25 pathways altered in co-ordinance with METTL3 and METTL14 (Fig. 3B, SF3A). Distinct pathways included apoptosis and stem cell pluripotency, and several cancer-related gene sets, such as melanoma and breast cancer (Fig. 3B, SF3D). Top Reactome gene sets included several immune regulatory pathways, altered by METTL3, METTL9, METTL13 and METTL14 either together or separately (Fig. 3C). METTL3, METTL13 and METTL14 knockdown all increased expression levels pro-inflammatory cytokines such as TNF, IL1A and CXCL8 (Fig. 3D) [49**–**51]. METTL13 knockdown uniquely increased expression of IL6, IL11 and IFNβ1a, which can inhibit cell cycle progression in hemopoietic malignances [52]. GSEA also showed an enrichment of many inflammatory KEGG pathways after knockdown of the METTL-genes (Fig. 3E). Lastly, METTL13 knockdown had the most exclusively dysregulated KEGG pathways, several crucial for malignant transformation, such as p53 signaling and pathways in cancer. Together, these data suggest that METTL13 may be uniquely disposed to promote survival, proliferation and possibly oncogenic transformation of HSPCs.

### METTL13 knockdown altered pathways involved in malignant transformation

**Fig. 4:**
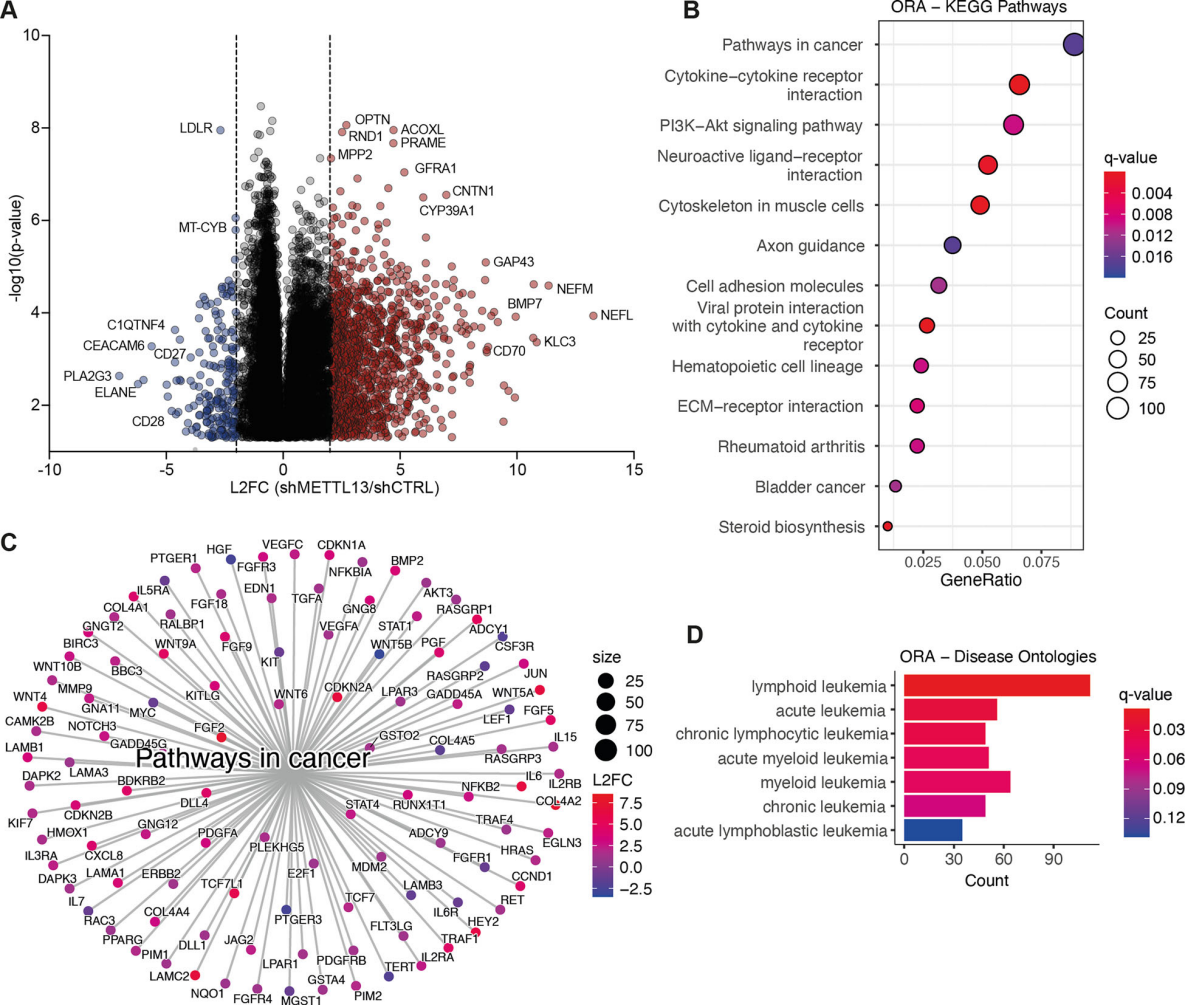
METTL13 knockdown caused dysregulation of pathways involved in malignant transformation. **A** Volcano plot of differentially expressed genes following METTL13 knockdown (shMETTL13, *n* = 4) compared to the control (shCTRL, *n* = 5) in human CD34^+^ HSPCs. Significance was calculated using unpaired two-tailed t-test, results are displayed as L2FC and negative log10 *p*-value (*p* < 0.05). **B** ORA of the top enriched KEGG pathways in shMETTL13 (*n* = 4) compared to shCTRL (*n* = 5). Created in R, with packages clusterprofiler and enrichPlot, statistics was set to: q.value < 0.1, L2FC cutoff = 1, p-adjust method = BH. **C** Network plot of the KEGG gene set pathways in cancer in shMETTL13 (*n* = 4) compared to shCTRL (*n* = 5), nodes are colored by L2FC. **D** ORA of disease ontologies focused on hematological malignances in shMETTL13 (*n* = 4) compared to shCTRL (*n* = 5). Created in R, with packages clusterprofiler, DOSE and enrichplot, statistics were set to: q.value ≤ 0.1, L2FC cutoff = 1, p-adjust method = BH.

The unique gene expression program following METTL13 knockdown prompted us to define METTL13´s role in HSPCs. Inhibition of METTL13 led to altered expression of many genes involved in important cellular processes, including an upregulation of CD70, part of the TNF superfamily, which together with CD27 plays a role in proliferation and survival (Fig. 4A, SF4A) [53]. METTL13 knockdown also downregulated CEACAM6, which regulates tumor proliferation and migration through ERK-MAPK and PI3K-AKT signaling in several cancers, including lung and colon cancer [54].

Over-representation analysis (ORA) revealed dysregulation of several KEGG pathways following METTL13 knockdown, both inflammatory pathways, including TNF signaling and cytokine-cytokine receptor interaction, and pathways regulating cell survival, such as PI3K-Akt signaling (Fig. 4B). The pathway with the greatest number of dysregulated genes was pathways in cancer, many of which are part of the WNT and NOTCH signaling pathways, crucial for oncogenic progression (Fig. 4B, C). ORA of METTL13 knockdown also revealed involvement of METTL13 in several disease ontologies, many related to infection and inflammation, like encephalitis and pneumonia (Fig. SF4B). METTL13 knockdown also altered genes belonging to disease ontologies involved in hematological malignancies, mainly lymphoid leukemias and acute leukemias (Fig. 4D). This strengthens our hypothesis that METTL13 might have distinct molecular functions leading malignant alterations in HSPCs, potentially inducing pre-leukemic transformation.

### Upregulation of METTL13 was associated with a high-risk profile in pediatric T-ALL

**Fig. 5:**
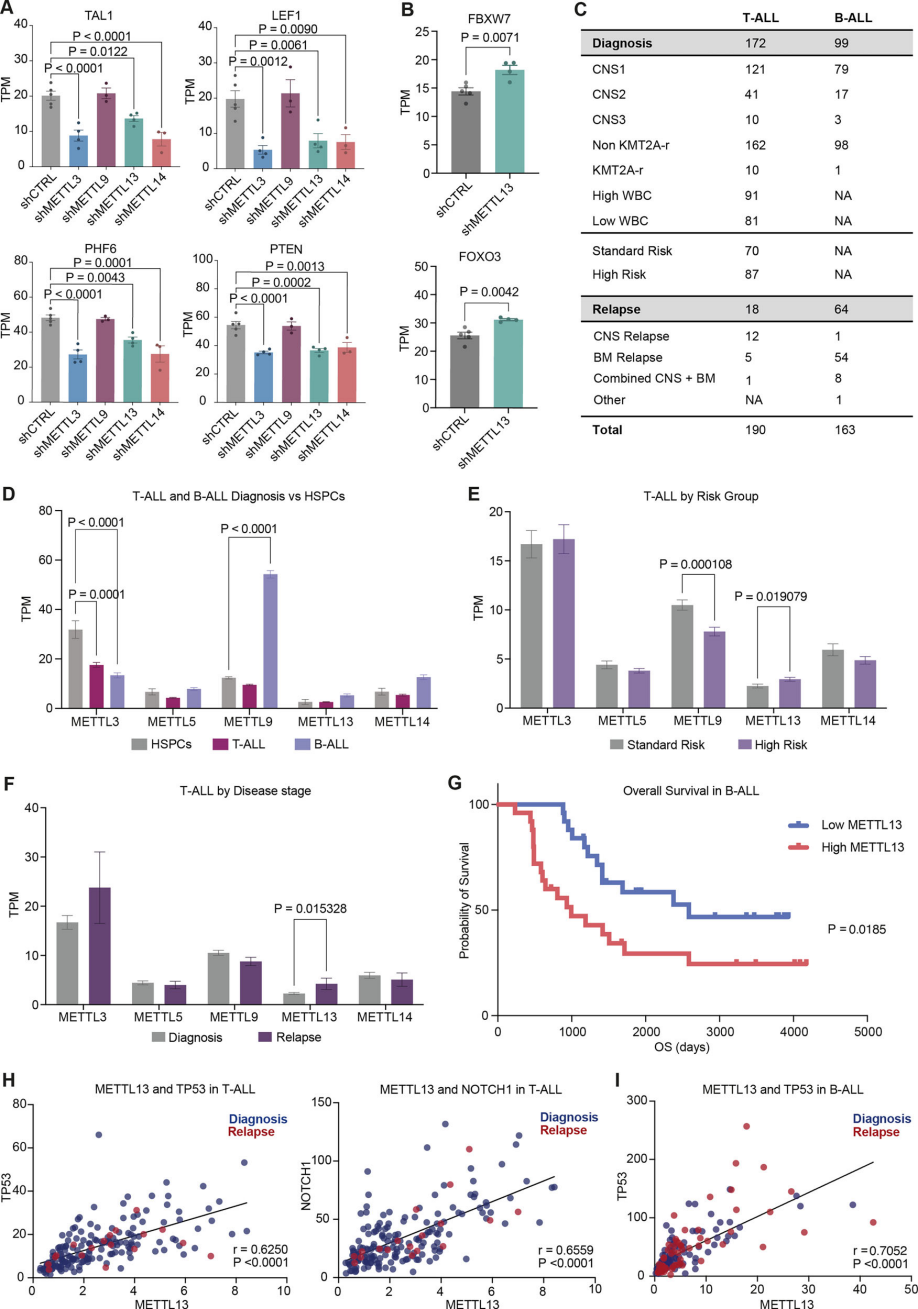
Upregulation of METTL13 was associated with a high-risk profile in pediatric T-ALL. **A** Dysregulated genes (TAL1, LEF1, PHF6 and PTEN) following METTL3 (*n* = 4), METTL9, (*n* = 3) METTL13 (*n* = 4) and METTL14 (*n* = 3) knockdown in human CD34^+^ HSPCs compared to the control (shCTRL, *n* = 5). Significance was calculated using ordinary one-way ANOVA with multiple comparisons compared to shCTRL, as well as Dunnett correction, results are displayed as TPM, mean ± SEM. **B** Uniquely dysregulated genes (FBXW7 and FOXO3) in shMETTL13 (*n* = 4) (not affected by METTL3, METTL9 or METTL14 knockdown) compared to shCTRL (*n* = 5). Significance was calculated using unpaired two-tailed t-test, results are displayed as TPM, mean ± SEM. **C** Table of ALL patient characteristics and subgroups used for RNA-sequencing analysis (publicly available by the TARGET Initiative). Samples were divided into T-ALL (*n* = 190) or B-ALL (*n* = 163), diagnosis or relapse, as well as different high-risk factors (CNS infiltration, KMT2A-r and WBC). **D** Expression levels of METTL3, METTL5, METTL9, METTL13 and METTL14 in T-ALL (*n* = 162) and B-ALL (*n* = 99) diagnosis samples (publicly available by the TARGET Initiative) compared to normal HSPCs (CD34 + CB, *n* = 5) obtained through RNA-sequencing. Significance was calculated using ordinary one-way ANOVA with multiple comparisons compared to HSPCs, as well as Dunnett correction. **E** Expression levels of METTL3, METTL5, METTL9, METTL13 and METTL14 in T-ALL patient samples (publicly available by the TARGET Initiative) generated through RNA-sequencing, displayed as TPM, mean ± SEM. T-ALL samples were grouped by risk stratification, into standard risk (CNS negative, non KMT2A-r and low WBC, *n* = 70), and high risk (CNS-infiltrated, KMT2A-r or high WBC, *n* = 87). Significance was calculated using multiple unpaired t-tests, corrected for multiple comparisons with the Holm-Šídák method. **F** Expression levels of METTL3, METTL5, METTL9, METTL13 and METTL14 in T-ALL patient samples (publicly available by the TARGET Initiative) generated through RNA-sequencing, displayed as TPM, mean ± SEM. T-ALL samples were grouped by disease stage, into diagnosis (standard risk, *n* = 87) and relapse samples (BM relapse, *n* = 5). Significance was calculated using multiple unpaired t-tests, multiple unpaired t-test, corrected for multiple comparisons with the Holm-Šídák method. **G** Survival probability in B-ALL patients samples (publicly available by the TARGET Initiative) by METTL13 expression levels (top and bottom 15%, *n* = 25 in each group). Significance was calculated using Log-rank (Mantel-Cox) test. **H** Correlation of METTL13 and TP53 as well as METTL13 and NOTCH1 in T-ALL patient samples, colored by the disease stage (diagnosis = blue, *n* = 162 and relapse = red, *n* = 18), results are displayed as TPM. Correlation was calculated using Pearson correlation coefficients with a two-tailed with 95% confidence interval. **I** Correlation of METTL13 and TP53 in B-ALL patient samples, colored by disease stage (diagnosis = blue (*n* = 99), relapse = red (*n* = 64)), results are displayed as TPM. Correlation was calculated using Pearson correlation coefficients with a two-tailed with 95% confidence interval.

Our HSPC profiling after METTL knockdowns suggests a distinct role for METTL13 in leukemic transformation. To further investigate this, we focused on pediatric ALL, including both T-ALL and B-ALL. Indeed, important oncogenes were downregulated following METTL3, METTL13 and METTL14 knockdown, such as TAL1, LEF1, PHF6, and PTEN (Fig. 5A) [55]. Similarly, knockdown caused a loss of ETV6 and CREBBP, candidate driver genes in B-ALL (Fig. SF5A). In contrast, both CDKN2A, a gene with tumor suppressive potential in both T-ALL and B-ALL, as well as KRAS, a B-ALL driver gene, were upregulated [56] (Fig. SF5A). Knockdown of all three METTLs caused a decreased expression of stem cell surface marker CD34 (Fig. SF5A) [57]. FBXW7 and FOXO3 were uniquely upregulated by the loss of METTL13, genes which have been identified as negative regulators of T-ALL progression, by inhibiting NOTCH1 activity and inducing apoptosis, respectively (Fig. 5B) [58, 59].

To evaluate the role of METTL-family members in pediatric ALL progression, we performed RNA-sequencing analysis on 190 T-ALL and 163 B-ALL samples from the TARGET initiative (Fig. 5C, Supplemental Table 3). We identified a significant loss of METTL3 in both T- and B-ALL samples compared to HSPCs (Fig. 5D). ALL samples were further grouped based on clinical risk factors, including white blood cell count (WBC), KMT2A-rearrangement (KMT2A-r) and central nervous system (CNS) infiltration [60]. Among T-ALL samples, we identified an upregulation of METTL13 in both high risk (CNS-infiltrated, KMT2A-r or a WBC above 100) and relapsed samples (with bone-marrow origin) compared to standard risk diagnosis samples (CNS negative, non-KMT2A-r, and a WBC below 100) (Fig. 5E, F). This type of risk stratification was not observed for other METTL genes, including METTL3 or METTL14. Although no significant difference in METTL13 expression was identified between B-ALL samples, low METTL13 was associated with a higher probability of survival (Fig. 5G, SF5B). METTL13 expression correlated positively with TP53 expression in both B- and T-ALL, as well as with NOTCH1 in T-ALL (Fig. 5h, I). Additionally, METTL13 expression had a significant positive correlation with ADAR1 in both the T- and B-ALL cohort (Fig. SF5C, SF5D). Interestingly, in METTL13 knockdown HSPCs, ADAR1 expression was upregulated (Fig. 2E), which likely represents a negative feedback loop between these two proteins. Our findings indicate converging functions of m6A writer genes METTL3 and METTL14, as well as METTL13 in pediatric ALL, while only METTL13 was associated with a high-risk profile in T-ALL and a decreased probability of survival in B-ALL.

### Loss of METTL13 impaired T-ALL proliferation and viability

**Fig. 6:**
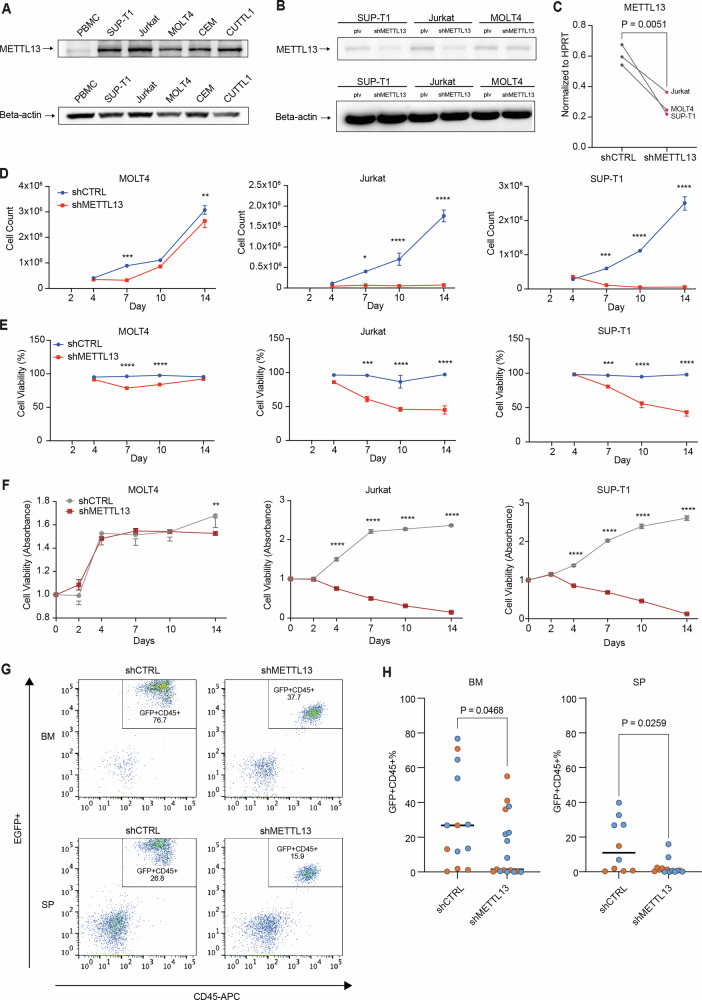
Loss of METTL13 impaired T-ALL cell proliferation and viability. **A** Western blot image showing the expression level of METTL13 and beta-actin in normal PBMCs and T-ALL cell lines (SUP-T1, Jurkat, MOLT4, CEM and CUTTL1). **B** Validation of METTL13 knockdown in T-ALL cell lines Jurkat, MOLT4 and SUP-T1 by western blot, showing the protein level of METTL13 and beta-actin, in cells transduced with an empty vector control (plv) or shMETTL13. **C** Validation of METTL13 knockdown (shMETTL13) in T-ALL cell lines Jurkat (*n* = 1), MOLT4 (*n* = 1) and SUP-T1 (*n* = 1) compared to the control (shCTRL, *n* = 1 for each cell line) through RT-qPCR. Significance was calculated using unpaired two-tailed t-test, results are displayed as expression level relative to the housekeeping gene (HPRT), mean ± SEM. **D** Total number of viable cells following METTL13 knockdown in T-ALL cell lines MOLT4 (*n* = 3), Jurkat (*n* = 3) and SUP-T1 (*n* = 3) from day 4 to day 14 post transduction compared to the control (*n* = 3 for each cell line). Significance was calculated using two-way ANOVA with Šídák’s multiple comparisons test, *p < 0.05, **p < 0.01, ***p < 0.001, ****p < 0.0001. **E** Cell viability (in percent) following METTL13 knockdown (Vector ID: VB240522-1552yyd) in T-ALL cell lines MOLT4 (*n* = 3), Jurkat (*n* = 3) and SUP-T1 (*n* = 3) from day 4 to day 14 post transduction compared to the control (*n* = 3 for each cell line). Significance was calculated using two-way ANOVA with Šídák’s multiple comparisons test, *p < 0.05, **p < 0.01, ***p < 0.001, ****p < 0.0001. **F** Cell viability was measured by MTT assay following METTL13 knockdown (Vector ID: VB240522-1553kfn) in T-ALL cell lines (*n* = 3 experimental triplicates) from day 2, 3, 7, 10 and 14 post-transduction. Absorbance was normalized to day 0. Significance was calculated using two-way ANOVA with Šídák’s multiple comparisons test, *p < 0.05, **p < 0.01, ***p < 0.001, ****p < 0.0001. **G** Representative flow cytometry showing human EGFP^+^CD45^+^ leukemia engraftment in NSG-SGM3 mice transplanted with SUP-T1 cells. **H** Engraftments of human EGFP^+^CD45^+^ were quantified by flow cytometry in bone marrow (BM) and spleen (SP) of SUP-T1 (blue) and MOLT4 (orange) transplanted mice (*n* = 10–17 mice per condition).

As the role and prognostic effect of METTL13 in pediatric leukemia is unexplored, we dived into the mechanistic link between METTL13 and leukemia propagation using T-ALL as a model. We first examined METTL13 expression in normal peripheral blood mononuclear cells (PBMCs) and five T-ALL cell lines using western blot (Fig. 6A, SF6A, uncropped western blots Fig. C). METTL13 was highly expressed in all T-ALL cell lines, with the highest expression in Jurkat, SUP-T1, and CUTTL1, followed by CEM and MOLT4. To directly examine the function of METTL13 in vitro, we knocked down METTL13 in three T-ALL cell lines with various level of METTL13 expression (SUP-T1, Jurkat and MOLT4). The knockdown was confirmed by both RT-qPCR and western blotting (Fig. 6B, C, SF6B, uncropped western blots Fig. D). METTL13 inhibition significantly reduced proliferation and survival starting from day 7 post-lentiviral transduction and persisted at 14 days post-transduction, both quantified by trypan-blue (Fig. 6D, E). The reduction in cell viability was further validated by another shRNA targeting METTL13 using MTT (3-(4,5- dimethythiazol-2-yl)-2,5-diphenyl tetrazolium bromide) assay (Fig. 6F). A stronger inhibition of cell viability and proliferation was found in high-METTL13-expressing cell lines (Jurkat and SUP-T1) compared to the relatively low-METTL13-expressing line (MOLT4), suggesting the cellular response may be dependent on cell-intrinsic METTL13 expression.

To validate the in vitro results, we also performed an in vivo cell line-derived xenograft (CDX) assay. We transduced SUP-T1 and MOLT4 cells with EGFP^+^ scramble control shRNA or a shRNA targeting METTL13 to track METTL13 knockdown. EGFP+ cells were transplanted into NSG-SGM3 immuno-compromised mice. The leukemia engraftment was detected in both spleen and bone marrow and reached as high as 80% in the control bone marrow (Fig. 6G, H). METTL13 knockdown significantly reduced the leukemia engraftment rate in bone marrow (29.9% in control and 14.4% in shMETTL13) and spleen (15.6% in control and 2.4% in shMETTL13) (Fig. 6H, mean values). Thus, we confirmed that METTL13 is important for leukemia proliferation in both in vitro and in vivo T-ALL models.

### Knockdown of METTL13 promoted pathways inducing apoptosis and suppressing DNA synthesis in T-ALL cells

We next investigated the effects of METTL13 inhibition on a transcriptional level in T-ALL by RNA-sequencing of MOLT-4, Jurkat, and SUP-T1 cell lines following METTL13 knockdown (Fig. SF7A). Our analysis revealed alterations to thousands of genes within each cell line (Fig. SF7B, Supplemental Table 4). The three T-ALL cell lines clustered separately in PCA with most differentially expressed genes unique to each cell line, indicating a greater variability between the cell lines, which likely represents the heterogenicity of T-ALL (Fig. SF7C). Despite this, all three cell lines also clustered into distinct METTL13 knockdown and control groups. A total of 1 529 significantly differentially expressed genes were shared among the cell lines, including 325 upregulated and 873 downregulated genes (Fig. SF7D–F).

**Fig. 7:**
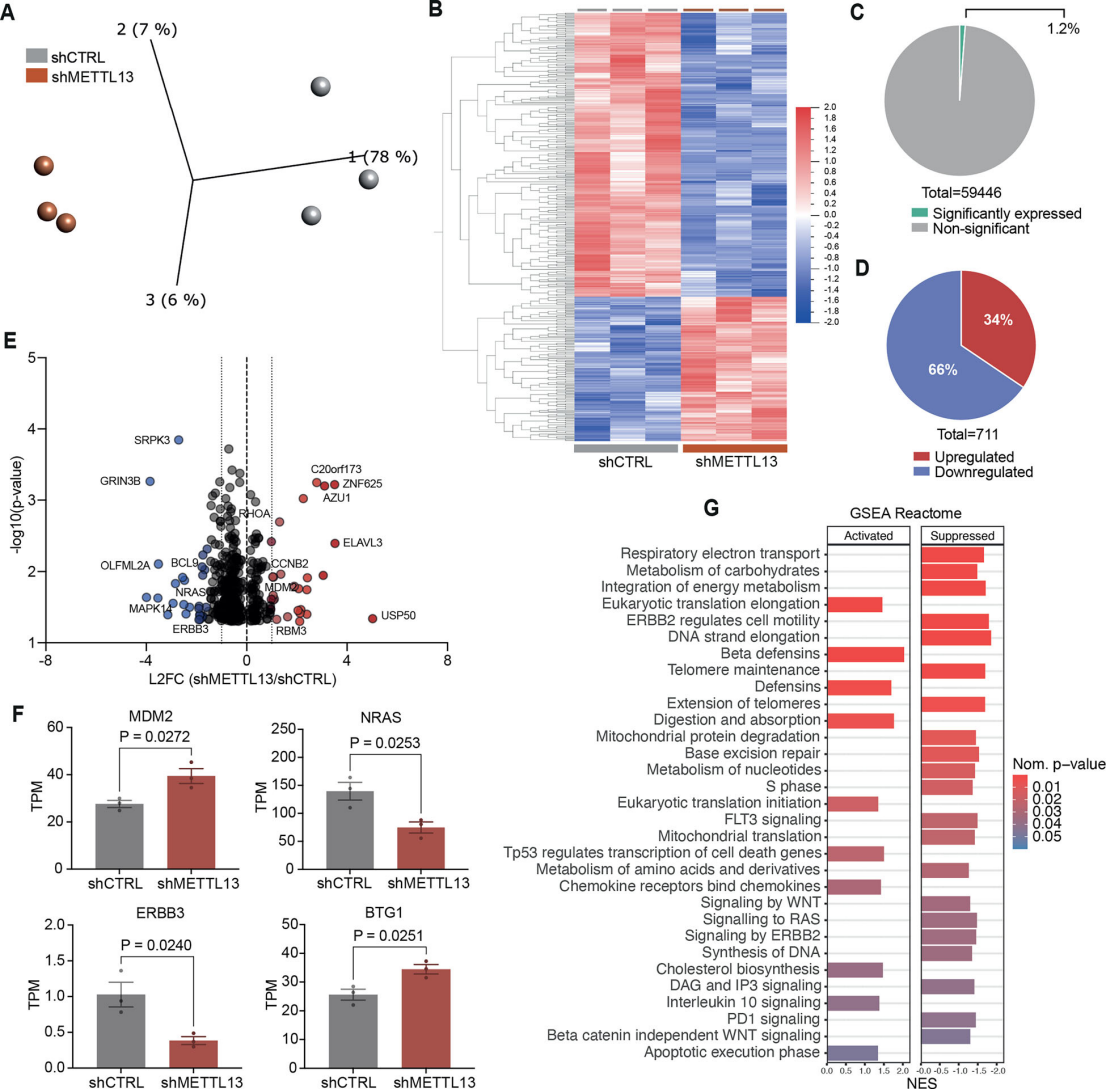
Knockdown of METTL13 promoted pathways inducing apoptosis and suppressing DNA synthesis in T-ALL cells. **A** PCA plot of T-ALL cell lines as biological replicates following METTL13 knockdown (shMETTL13, *n* = 3) compared to the control (shCTRL, *n* = 3). Significance was calculated using unpaired two-tailed t-test, *p* < 0.05. **B** Heat map of the top 400 differentially expressed genes looking at T-ALL cell lines as biological replicates in shMETTL13 (*n* = 3) compared to the shCTRL (*n* = 3). Significance was calculated using unpaired two-tailed t-test, *p* < 0.05. **C** Pie chart of the percentage of significantly expressed genes (*p* < 0.05) in T-ALL cell lines as biological replicates (*n* = 3) in shMETTL13 (*n* = 3) compared to shCTRL (*n* = 3). Significance was calculated using unpaired two-tailed t-test, *p* < 0.05. **D** Pie chart of the distribution of upregulated versus downregulated significantly expressed genes in T-ALL cell lines as biological replicates in shMETTL13 (*n* = 3) compared to the shCTRL (*n* = 3). Significance was calculated using unpaired two-tailed t-test, *p* < 0.05. **E** Volcano plot of significantly expressed genes (*p* < 0.05) in T-ALL cell lines as biological replicates in shMETTL13 (*n* = 3) compared to the shCTRL (*n* = 3). Only protein-coding genes were included in this plot. Significance was calculated using unpaired two-tailed t-test, results are displayed as L2FC and negative log10 *p*-value (*p* < 0.05). **F** Dysregulated genes (MDM2, NRAS, ERBB3 and BTG1) in shMETTL13 (*n* = 3) compared to shCTRL (*n* = 3). Significance was calculated using unpaired two-tailed t-test, *p* < 0.05, results are displayed as TPM, mean ± SEM. **G** GSEA of some of the top significantly enriched Reactome pathways (Created in GSEA and MSigDB, nominal *p*-value < 0.05) in shMETTL13 (*n* = 3) compared to shCTRL (*n* = 3) in T-ALL cells, using only protein-coding genes, displayed as activated (positive NES) or suppressed (negative NES).

To counteract the cell-intrinsic differences among cell types, the cell lines were treated as biological replicates, divided into shMETTL13 and shCTRL. PCA showed a clear separation between the two conditions, also visualized by the gene expression pattern (Fig. 7A, B). Approximately 1% of all genes were differentially expressed, most being downregulated (Fig. 7C, D). METTL13 knockdown inhibited B-ALL driver gene NRAS, and ERBB3, which is frequently overexpressed in cancer (Fig. 7E, F) [55, 61]. Upregulated genes included MDM2 and BTG1, which has been identified to induce cell cycle arrest and inhibit cell proliferation in several different malignancies [62]. Furthermore, GSEA revealed that knockdown of METTL13 suppressed nucleotide metabolism, WNT and RAS signaling, as well as DNA synthesis. Contrarily, METTL13 knockdown activated TP53 regulation of cell death genes, IL10 signaling and apoptotic execution phase (Fig. 7G). The results from the pathway analysis, together with both the in vitro and in vivo validation, highlight the potential impact METTL13 could have on leukemia cell survival and proliferation. Collectively, these data indicate a possible oncogenic role of METTL13 in pediatric T-ALL pathogenesis.

## Discussion

To better understand how RNA modifications contribute to leukemic transformation, we wanted to investigate the transcriptional role of members from the METTL-family and ADAR1, in the context of pre-leukemic transformation and pediatric leukemia. Previous studies have shown that RNA methylation by the m6A complex increases RNA editing, by enhancing ADAR1 levels in response to IFN stimulation [48], and METTL3 was found to increase protein levels of ADAR1 in glioblastoma [63]. Notably, this regulatory relationship appears to be context dependent. In contrast to reports in glioblasoma linking METTL3 activity to altered ADAR1 protein abundance, we observed no change in ADAR1 expression levels following METTL3 or METTL14 knockdown, and no change in ADAR1 protein levels following METTL13 knockdown in T-ALL cell lines, despite the transcriptional upregulation observed in cord blood HSPCs. These findings suggests that METTL13-dependent phenotypes in T-ALL may occur through mechanisms that are independent of ADAR1 protein abundance and reflect fundamental differences between normal hematopoietic progenitors and malignant lymphoid cells. In contrast, ADAR1 was found to preferentially bind to m6A-depleted RNA transcripts, indicating a negative correlation between A-to-I editing and m6A [64]. In our dataset, ADAR1 overexpression was accompanied by reduced transcription of several m6A associated genes (METTL3 and METTL14, as well as erasers FTO and ALKBH5). An orphan METTL-family member, METTL13, was selectively upregulated by ADAR1. Interestingly, METTL13 was upregulated by ADAR1 overexpression, of both wild-type ADAR1 and an editing-defective ADAR1 mutant, suggesting that ADAR1 could alter METTL13 expression through editing-independent functions. These findings position METTL13 within a transcriptional program that intersects with ADAR1-driven pathways. Further work will be required to determine the direct functional relationship between m6A and A-to-I RNA editing using m6A-seq and RNA editome analysis in the context of HSPCs.

We next compared the distinct role of METTL13 to other METTL-family members in HSPCs by systematical knockdown of each METTL individually. These studies revealed that many differentially expressed genes were regulated both by the core members of the m6A writer complex (METTL3 and METTL4) and by METTL13, suggesting that METTL13 could act in convergence with the m6A writer complex. Although many pathways were altered by METTL13 as well as METTL3 and METTL14, several pathways were uniquely affected by METTL13 knockdown, including p53 signaling and cancer-associated pathways. Knockdown of METTL-family members produced transcriptome coordinate changes in several canonical regulators of cell survival and stress, including reduced TP53 and increased MDM2 expression, alongside an upregulation of CDKN1A and suppression of apoptotic genes (CASP3, DFFB) and c-MYC. Although some of these transcriptional responses appear divergent, such mixed patterns are not unexpected when perturbing RNA-modifying enzymes with widespread influence on RNA stability, splicing, and translation. These factors regulate hundreds of transcripts simultaneously, and their depletion can trigger compensatory or context-dependent stress programs rather than a single linear pathway. For example, simultaneous induction of MDM2 (pro-proliferative) and BTG1 (cell-cycle inhibitory) following METTL13 knockdown in T-ALL cell lines likely reflect parallel engagement of checkpoint pathways and adaptive feedback loops, rather than mutually exclusive biological outcomes. Thus, these gene-expression signatures should be interpretated as reflecting broad network-level responses to METTL perturbation, rather than as evidence for a single defined mechanistic cascade. Future studies that rescue individual genes, including METTL13, in the presence of METTL13 knockdown will further elucidate complex molecular pathways.

Our findings highlight potential effects of METTL13 in malignant transformation, which prompted further analysis to unravel its specific role in hematological diseases. We utilized publicly available RNA-sequencing datasets from the TARGET initiative, including both pediatric T and B-ALL samples. A recent study described elevated METTL3 levels in pediatric B-ALL relapse samples when compared with adult peripheral blood and bone marrow from patients with other malignancies [65]. Surprisingly, we found that METTL3 was expressed at lower levels in both T and B-ALL compared to HSPCs. Differences in comparator tissues, age groups, and cellular composition likely contribute to divergent patterns. Our findings also reveal a reduced survival probability in pediatric B-ALL patients with high METTL13 expression, and METTL13 was overexpressed in both high-risk and relapsed T-ALL. Finally, METTL knockdown reduced survival and proliferation of T-ALL cells in vitro and in vivo. Knockdown of METTL13 significantly reduced the leukemia engraftment rate in the CDX model. RNA-sequencing analysis of T-ALL cells with depleted METTL13 expression revealed activation of p53 signaling and apoptosis, while inhibiting DNA synthesis and signaling pathways (WNT and RAS signaling), crucial for cell proliferation and leukemic progression.

Of note, METTL13 knockdown in HSPCs altered genes involved in inflammatory, WNT, and NOTCH signaling and reduced TP53 expression. In contrast, knockdown of METTL13 in T-ALL cell lines triggered TP53-dependent apoptosis and high METTL13 levels were associated with high-risk disease and poorer survival in pediatric T-ALL. The different signatures likely reflect distinct adaptive programs in normal HSPCs versus transformed T-ALL cells. Similarly, in T-ALL cell lines the magnitude of the response to METTL13 knockdown reflected each cell line’s intrinsic METTL13 expression level, with a stronger inhibition of cell viability and proliferation in high-METTL13-expressing cell lines. These findings together indicate that METTL13 most likely functions in a context-dependent manner, with transcriptional outputs shaped by cellular state, lineage, and oncogenic background.

Recent studies have identified METTL13 as a lysine methyltransferase that promotes translation and have started to unravel the role of METTL13 in oncogenic development. [33**–**36]. However, research on METTL13 and its role in HSC biology and leukemia is sparse. Our findings align with emerging work by Zhao et al. (2025) who highlighted that METTL13-driven MYC programs contribute to malignant progression in AML, and their results similarly highlight METTL13 as a regulator of oncogenic behavior [37]. Together with our data in HSPCs and T-ALL this strengthens the concept that METTL13 acts at multiple post-transcriptional levels to support leukemic survival and proliferation. However, the precise mechanistic relationship between METTL13’s translational role and the transcriptional programs we identify here remains to be determined.

In conclusion, the collective findings following METTL13 knockdown in HSPCs and T-ALL cells suggest that METTL13 may be involved in promoting pre-leukemic development of HSPCs and in the pathogenesis of T-ALL. Future studies are necessary to functionally determine effects of METTL13 in leukemic progression, yet our findings highlight a novel, distinct role of METTL13 in T-ALL development.

## Materials and methods

### Cord blood and cell line handling

CD34^+^ human cord blood samples and PBMCs were purchased from commercial vendors (StemCell Technology or AllCells) and stored in liquid nitrogen until ready for use. T-ALL cell lines (CEM, CVCL_0207; CUTTL1, CVCL_4966; MOLT-4, RRID:CVCL_A1BB; SUP-T1, RRID:CVCL_1714; and Jurkat, RRID:CVCL_0065) were purchased from ATCC and maintained in RPMI media (Gibco, 11875119) supplemented with 10% FBS (Gibco) and 1% penicillin-streptomycin (Gibco, 15140) at 37 °C in 5% CO2.

### Lentiviral construct and transduction

To determine the function of members of the METTL-family, lentiviral knockdown in HSPCs and T-ALL cells was performed. Lentiviral construct of shRNAs targeting METTLs was purchased from VectorBuilder (Vector ID: VB240522-1552yyd and VB240522-1553kfn). All lentivirus was tested by transducing HEK293T cells, and the knockdown efficiency and titers were assessed by FACS analysis of GFP signal and RT-qPCR of the target genes. Cord blood CD34^+^ cells were cultured in 96-well plate (5 ×10^5^ cells per well) containing StemPro (Life Technologies) media supplemented with human cytokines (IL-6, stem cell factor (SCF), Thrombopoietin (Tpo) and FLT-3, all from R&D Systems) for 2-3 days at lentivirus at a MOI of 100–200. T-ALL cell lines were cultured in 24-well plate or 6-well plate (5–10 ×10^5^ cells per well) in culture media with lentivirus at a MOI of 5-10. Cells were then collected for downstream analysis.

### Animal experiments and flow cytometry analysis

All mouse studies were conducted under protocols approved by the Institutional Animal Care and Use Committee (IACUC) of the University of California, San Diego and were in compliance with federal regulations regarding the care and use of laboratory animals: Public Law 99-158, the Health Research Extension Act, and Public Law 99-198, the Animal Welfare Act which is regulated by USDA, APHIS, CFR, Title 9, Parts 1, 2, and 3. Immunocompromised NSG-SGM3 mice were bred and maintained in the Sanford Consortium for Regenerative Medicine vivarium according to IACUC approved protocols of the University of California, San Diego. Neonatal mice of both sexes were used in the study. MOLT4 or SUP-T1 cells were injected intrahepatically into 2-3 days old neonatal NSG-SGM3 mice at 2.5-5×10^4^ cells per pup. Leukemic engraftment was quantified by FACS analysis-based peripheral blood screening of human CD45^+^ population starting from week 3 for every week until the engraftment reaches 10%. Mice were humanely sacrificed at 4-8 weeks, and cells were collected from hematological organs (bone marrow and spleen) for FACS analysis. Cells were resuspended in staining media (ice cold DPBS with 2% FBS), followed by blocking using FcR block (Cat 130-059-901, Miltenyi Biotech) for 15 min. CD45-APC cell surface antibody (Cat # 304037, Biolegend, San Diego, CA) was added a final dilution of 1:25 and incubated on ice for 30 min in the dark. DAPI solution was added before analysis to exclude dead cell debris. Flow analysis was performed on BD Aria Fusion, Aria II. Sample sizes (*n* = 10–17 mice per condition) were chosen based on established practices for T-ALL xenograft models and prior experience; and to account for neonatal mortality prior to leukemia engraftment. No randomization was performed. Neonatal mice of both sexes were used and assigned to experimental groups based on cell line and shRNA condition. Investigators were not blinded to group allocation during experiments or outcome assessment.

### Western blot analysis

Cells were collected and washed three times with cold PBS and resuspended in RIPA buffer supplemented with protease and phosphatase inhibitors and quantified by BCA assay. Protein lysate (10 μg) was separated by 10% SDS–PAGE and transferred to PVDF membranes. The membrane was blocked in 5% BSA/20 mM Tris-HCL for 30 min while rocking and probed with primary antibodies (METTL13 antibody, abcam, ab186002; beta-actin antibody, Millipore Sigma, A2228; ADAR1 antibody, Cell Signaling, 14175) overnight at 4 °C followed by incubation with HRP-conjugated secondary antibodies for 2 h at room temperature. Signals were visualized using SuperSignal West Femto Substract (ThermoFish, #34096) on a ChemiDoc System (Bio-rad).

### RNA extraction and quantitative real-time polymerase chain reaction (RT-qPCR)

For validation of lentiviral knockdown, RNA was extracted and analyzed through RT-qPCR. RNA extraction was performed using RNeasy micro extraction kits (QIAGEN) following the manufacturer’s protocol. A DNase incubation step was included to digest any genomic DNA. RNA (100-1000 ng) was converted to cDNA using the Super-Script III kit (ThermoFisher Scientific) according to the manufacturer’s recommended protocol. qRT-PCR was performed using SYBR GreenER Super Mix (Life Technologies) on BioRad CFX382 with 5 ng of template mRNA and 0.2 μM of each forward and reverse primer. Target mRNA was normalized to hypoxanthine phosphoribosyl transferase (HPRT) mRNA transcript levels and fold change was calculated via the delta-delta cycle threshold (CT) method. RT-qPCR primers are listed in Supplemental Table 5.

### Viability and proliferation analysis

To define effects of METTL13 knockdown on T-ALL cells, T-ALL cell lines were transduced with lentiviral scramble control or shRNA targeting shMETTL13 at a low MOI of 10–20 in culture media without pen/strep. After 2-3 days, cells were plated in 96-well plates at 1 ×10^4^ cells per well. At each time point, cell number and viability were determined by trypan blue assay.

The 3-(4,5- dimethythiazol-2-yl)-2,5-diphenyl tetrazolium bromide (MTT) assay was performed (Millipore, #11465007001) by seeding the transduced cells into a 96-well plate at a density of 1 ×10^4^ cells per well and the cell viability were determined at various time points. At each time point, 10 µL of MTT solution (5 mg/mL in PBS) was added per well, followed by a 4-h incubation at 37 °C. Subsequently, 100 µL of stabilizing reagent was added and the absorbance was quantified at 570 nm. Cell viability was calculated as a fold change to absorbance at day 0 timepoint.

### RNA-sequencing following lentiviral knockdown

The RNA-sequencing dataset transduced with lentiviral backbone (pCDH), wildtype ADAR1 (isoform p150), and editing defective ADAR1 mutant data (ADAR1^E912A^) were available from previous studies and processed and normalized as described in the previous publication (BioProject: PRJNA319866) [6, 8]. For RNA-sequencing of METTL knockdown in HSPCs and T-ALL, samples with RNA integrity numbers (RIN) ≥ 7 were proceeded for bulk RNA-sequencing. RNA-sequencing of HSPCs transduced with METTL3, METTL5, METTL9, METTL13 and METTL14 knockdown was performed using 50–100 ng of RNA by Scripps Research on SMARTer seq system, with paired-end 150 reads, at 50 million reads per sample. Obtained reads were aligned using STAR two-pass alignment method and the reference genome GRCh38.84 together with the corresponding GFT file, to generate transcriptome-coordinate based BAM files as described in a previous study [10]. Raw counts were obtained through STAR, using the ENCODE STAR-RSEM pipeline generating the numbers of reads aligned to each gene. Transcripts per million (TPM) values were calculated over the total collapsed exonic regions for each gene. Based on the PCA analysis, three outlier samples were excluded from further experimental analysis. RNA-sequencing of T-ALL cells transduced with METTL13 knockdown was performed using 100 ng of RNA by Novogene on NovaSeq X Plus sequencing system with paired-end 150 reads. Raw counts were generated from BAM files together with the provided GTF file in R (version 4.3.1) using FeatureCount from the Rsubread package (version 2.16.1) with the pipeline set to unstranded and paired end reads. Raw count data was normalized in R (version 4.3.1) to TPM using the mRNA expression transformation guideline provided by the GDC bioinformatics pipeline. All RNA-sequencing experiments were conducted in triplicates.

### Publicly available RNA-sequencing downloading and normalization

RNA-sequencing data from pediatric T-ALL patients was obtained from publicly available data generated by the TARGET initiative. The RNA-sequencing dataset (.BAM) were acquired via the GDC Data Portal (https://portal.gdc.cancer.gov/projects/TARGET-ALL-P2). A total of 190 bone marrow-derived T-ALL samples (192 from time of diagnosis, 18 after relapse, non-longitudinal) were included. Sample inclusion was based on the sample type (bone marrow), disease status (diagnosis and relapse) as well as MLL status (positive or negative, samples with status unknown were excluded). Raw counts were generated using the SeqMonk Mapped Sequence Data Analyzer tool (version 1.47.1). The RNA-seq quantitation pipeline was set to follow the subsequent criteria: transcript features were set to mRNA, library type was selected as opposing strand specific and transcript isoforms were merged. Raw count data from CD34^+^ cord blood was obtained from the publicly available data set found in the Gene Expression Omnibus (GEO) at GSE190269 [66]. Raw count data was normalized in R (version 4.3.1) to TPM using the mRNA expression transformation guideline provided by the GDC bioinformatics pipeline. RNA-sequencing from pediatric B-ALL patients was generated by the TARGET initiative and obtained from the cBioPortal for Cancer Genomics [67**–**69]. A total of 163 bone marrow-derived samples were included (99 from the time of diagnosis, 64 after relapse, non-longitudinal). The data was downloaded as RPKM and converted to TPM (TPM = (RPKM x 10^6^) / (sum of all RPKM values in the sample)) in R (version 4.3.1).

### RNA quantification and functional enrichment

Subsequent analysis was based on normalized RNA-sequencing data (TPM), generated from raw counts as described above. PCA and heatmaps were created using Qlucore Omics Explorer (version 3.9). Significance for the heatmap was calculated using multi-group ANOVA (*q* < 0.1, SD < 0.05). ORA was performed by including all significantly differentially expressed genes (calculated using unpaired two-tailed t-test, *p* < 0.05) and generated in R (version 4.3.1), using packages ClusterProfiler (version 4.8.3), DOSE (version 3.28.2) and Enrichplot (version 1.20.3). For ORA, p-adjust method was set to BH and L2FC cutoff of 1. GSEA was performed using GSEA software (version 4.3.2) provided by Broad Institute and UC San Diego [70, 71], utilizing human KEGG Legacy (version 2024.1.Hs), Reactome (version 2024.1.Hs) and Wiki Pathway (version 2024.1.Hs) gene sets. For GSEA, only protein-coding genes were included. For analysis of differentially expressed genes, genes with a L2FC = NA were excluded. Differentially expressed genes were obtained using unpaired two-tailed t-test between the different experimental conditions compared to the control group (*p* < 0.05).

### Statistical analysis

Statistical analyses were performed using ordinary one-way ANOVA of the mean of every condition against the control group with multiple comparisons (using normalized TPM values), applying Dunnett correction, using unpaired two-tailed t-test when comparing normalized TPM values between two groups, multiple unpaired t-tests corrected for multiple comparisons with the Holm-Šídák method or two-way ANOVA with Šídák’s multiple comparisons. Correlation were assessed using Pearson correlation coefficients (two-tailed, 95% confidence interval). Analysis of cell viability and proliferation following in vitro experiments was performed using ordinary two-way ANOVA with multiple comparisons, and analysis of cell engraftment in vivo was performed using Mann-Whitney test. Statistical analyses were conducted using GraphPad Prism (v9) or R (version 4.3.1). Variance and distribution assumptions were addressed by using parametric or non-parametric tests as appropriate. Error bars represents mean ± SEM. Significance was set to *P* < 0.05.

## Supplementary information

Supplemental Figure and Table Legend

Supplemental Figure 1

Supplemental Figure 2

Supplemental Figure 3

Supplemental Figure 4

Supplemental Figure 5

Supplemental Figure 6

Supplemental Figure 7

Supplemental Table 1

Supplemental Table 2

Supplemental Table 3

Supplemental Table 4

Supplemental Table 5

Uncropped western blots

## Data Availability

The RNA-sequencing dataset used in this study will be uploaded to an appropriate data portal upon request with a special password for editors and reviewers. The data will be made publicly available upon acceptance for publication. Further information and requests for resources and reagents should be directed to and will be fulfilled by Dr. Frida Holm (frida.holm@ki.se).
