# Supplementary material for: Uncovering a role for METTL13 in malignant transformation of human hematopoietic stem cells and in the progression of pediatric leukemia
Source: Cell Death Dis. 2026 Apr 25;17(1):549. doi: 10.1038/s41419-026-08761-7 (PMC13243463; doi:10.1038/s41419-026-08761-7)
Supplement: Supplementary file 1 — Supplemental Figure and Table Legend [file 41419_2026_8761_MOESM1_ESM.docx]

**Supplemental Figure and Table Legend**

**Supplemental Figure 1.**

A) Validation of ADAR1 overexpression in human CD34^+^ HSPCs (n=3) compared to the backbone control (pCDH, n=3). Significance was calculated using unpaired two-tailed t-test, results are displayed as TPM, mean ± SEM.

B) Expression levels of genes in the METTL-family following ADAR1 overexpressed cells (n=3) compared to the pCDH control (n=3). Significance was calculated using unpaired two-tailed t-test, results are displayed as TPM, mean ± SEM.

C) Expression of significantly altered genes (p < 0.05) from the m6A complex, METTL-family and ADAR1 in HSPCs overexpressed with an editing defective ADAR1 mutant (n=3) compared to the pCDH control (n=3). Significance was calculated using unpaired two-tailed t-test, results are displayed as L2FC of TPM values.

**Supplemental Figure 2.**

A) Validation of METTL knockdown (shMETTL3 (n=3), sMETTL5 (n=3), shMETTL9 (n=3), shMETTL13 (n=3) and shMETTL14 (n=3)) in human CD34^+^ HSPCs compared to the control (shCTRL, n=3) through RT-qPCR. Significance was calculated using unpaired two-tailed t-test,

results are displayed as expression level relative to the housekeeping gene (HPRT), mean ± SEM.

B) Validation of METTL knockdown (shMETTL3 (n=4), sMETTL5 (n=2), shMETTL9 (n=3), shMETTL13 (n=4) and shMETTL14 (n=3)) in HSPCs compared to the control (shCTRL, n=5)

through RNA-sequencing. Significance was calculated using unpaired two-tailed t-test, results are displayed as TPM, mean ± SEM.

C) Western blot image showing protein levels of METTL13 in CD34^+^ HSPCs transduced with an scramble control vector (plv) or shMETTL13 lentivirus.

D) Raw western blot image (panel C) showing protein levels of METTL13 in CD34+ HSPCs transduced with an empty vector (plv) or shMETTL13.

E) PCA plot of shMETTL3 (n=4), shMETTL5 (n=2), shMETTL9 (n=3), shMETTL13 (n=4) and shMETTL14 (n=3) in HSPCs compared to shCTRL (n=5) with the outliers labeled (excluded in sequential analysis). Created in Qlucore Omics Explorer.

F) Percentage of up- and downregulated genes (p<0.05) in shMETTL3 (n=4), shMETTL9 (n=3), shMETTL13 (n=4) and shMETTL14 (n=3) in HSPCs compared to shCTRL (n=5). Significance was calculated using unpaired two-tailed t-test.

G) Western blot image showing protein levels of ADAR1 (p110 and p150 isoforms) and beta-actin in T-ALL cell lines (SUP-T1, Jurkat, and MOLT4) transduced with the empty vector (plv) or shMETTL13.

H) Raw western blot image (panel G) showing protein levels of ADAR1 (p110 and p150) and beta-actin in T-ALL cell lines (SUP-T1, Jurkat, and MOLT4) transduced with the empty vector (plv) or shMETTL13.

**Supplemental Figure 3.**

A) Venn diagram of GSEA Wiki Pathways between shMETTL3 (n=4), shMETTL9 (n=3), shMETTL13 (n=4) and shMETTL14 (n=3) in human CD34^+^ HSPCs compared to shCTRL (n=5) (FDR q<0.1).

B) Venn diagram of GSEA Reactome pathways between shMETTL3 (n=4), shMETTL9 (n=3), shMETTL13 (n=4) and shMETTL14 (n=3) compared to shCTRL (n=5) (FDR q<0.1).

C) Venn diagram of GSEA KEGG pathways between shMETTL3 (n=4), shMETTL9 (n=3), shMETTL13 (n=4) and shMETTL14 (n=3) compared to shCTRL (n=5) (FDR q<0.1).

D) Bar plot of all unique GSEA Wiki Pathways (FDR.q <0.1) in shMETTL13 (n=4) compared to shCTRL (n=5). Results are displayed by NES.

**Supplemental Figure 4.**

A) Top differentially expressed genes following METTL13 (shMETTL13, n=4) knockdown in human CD34^+^ HSPCs compared to control (shCTRL, n=5). Significance was calculated using unpaired two-tailed t-test (p < 0.05), results are displayed as L2FC (shMETTL13/shCTRL).

B) ORA of top disease ontologies in shMETTL13 (n=4) compared to shCTRL (n=5). Created in R, packages ClusterProfiler, DOSE and Enrichplot, with statistics set to: q.value ≤ 0.1, L2FC cutoff = 1, p-adjust method = BH.

**Supplemental Figure 5.**

A) Dysregulated genes (ETV6, CREBBP, KRAS, CD34 and CDKN2A) METTL3 (shMETTL3, n=4), METTL9, (shMETTL9, n=3) METTL13 (shMETTL13, n=4) and METTL14 (shMETTL14, n=3) knockdown in human CD34^+^ HSPCs compared to the control (shCTRL, n=5). Significance was calculated by ordinary one-way ANOVA with multiple comparisons compared to shCTRL, as well as Dunnett correction, results are displayed as TPM, mean ± SEM.

B) Expression levels of METTL3, METTL5, METTL9, METTL13 and METTL14 in B-ALL patient samples (publicly available by the TARGET Initiative) generated through RNA-sequencing. B-ALL samples were grouped by disease stage, into diagnosis (n=99) or relapse samples (n=64), samples are longitudinal. Significance was calculated using multiple unpaired t-test, corrected for multiple comparisons with the Holm-Šídák method, results are displayed as TPM, mean ± SEM.

C) Correlation of METTL13 and ADAR1 in T-ALL patient samples, colored by the disease stage (diagnosis = blue, n=162 and relapse = red, n=18), results are displayed as TPM. Correlation was calculated using Pearson correlation coefficients with a two-tailed with 95% confidence interval.

D) Correlation of METTL13 and ADAR1 in B-ALL patient samples, colored by disease stage (diagnosis = blue (n=99), relapse = red (n=64)), results are displayed as TPM. Correlation was calculated using Pearson correlation coefficients with a two-tailed with 95% confidence interval.

**Supplemental Figure 6.**

A) Full length raw western blot image showing the expression level of METTL13 and beta-actin in normal PBMCs and T-ALL cell lines (SUP-T1, Jurkat, MOLT4, and CEM).

B) Full length raw western blot image showing the expression level of METTL13 and beta-actin in T-ALL cell lines (SUP-T1, Jurkat, and MOLT4) transduced with the empty vector (plv) or shMETTL13.

**Supplemental Figure 7.**

A) Validation of METTL13 knockdown (shMETTL13) in T-ALL cell lines Jurkat (n=3), MOLT4 (n=3) and SUP-T1 (n=3) compared to the control (shCTRL, n=3 for each cell line) through RNA-

sequencing. Significance was calculated using unpaired two-tailed t-test, results are displayed as TPM, mean ± SEM.

B) Distribution of differentially expressed genes in shMETTL13 compared to shCTRL in each T-ALL cell line separately (n=3 for each cell line and condition). Significance was calculated using unpaired two-tailed t-test, p < 0.05.

C) PCA plot of shMETTL13 compared to shCTRL in T-ALL cell lines Jurkat, MOLT4 and SUP-T1 (n=3 for each cell line and condition). Created in Qlucore Omics Explorer.

D) Venn diagram of all differentially expressed genes (p<0.05) in shMETTL13 compared to shCTRL in each T-ALL cell line separately (n=3 for each cell line and condition). Significance was calculated using unpaired two-tailed t-test, p < 0.05.

E) Venn diagram of significantly upregulated genes (p<0.05) in shMETTL13 compared to shCTRL in each T-ALL cell line separately (n=3 for each cell line and condition). Significance was calculated using unpaired two-tailed t-test, p < 0.05.

F) Venn diagram of significantly downregulated genes (p<0.05) in shMETTL13 compared to shCTRL in each T-ALL cell line separately (n=3 for each cell line and condition). Significance was calculated using unpaired two-tailed t-test, p < 0.05.

**Supplemental Tables**

**Supplemental Table 1.** Differentially expressed genes following ADAR1 overexpression in HSPCs (ADAR1 wild-type in tab 1, editing-defective ADAR1 mutant in tab 2)

**Supplemental Table 2.** Differentially expressed genes following METTL3, METTL5, METTL9, METTL13 and METTL14 knockdown in HSPCs

**Supplemental Table 3.** Clinical characteristics of ALL patient samples provided by TARGET

**Supplemental Table 4.** Differentially expressed genes following METTL13 knockdown in T-ALL cell lines

**Supplemental Table 5.** RT-qPCR Primers
