# Supplementary figures and images for: Uncovering a role for METTL13 in malignant transformation of human hematopoietic stem cells and in the progression of pediatric leukemia
Source: Cell Death Dis. 2026 Apr 25;17(1):549. doi: 10.1038/s41419-026-08761-7 (PMC13243463; doi:10.1038/s41419-026-08761-7)

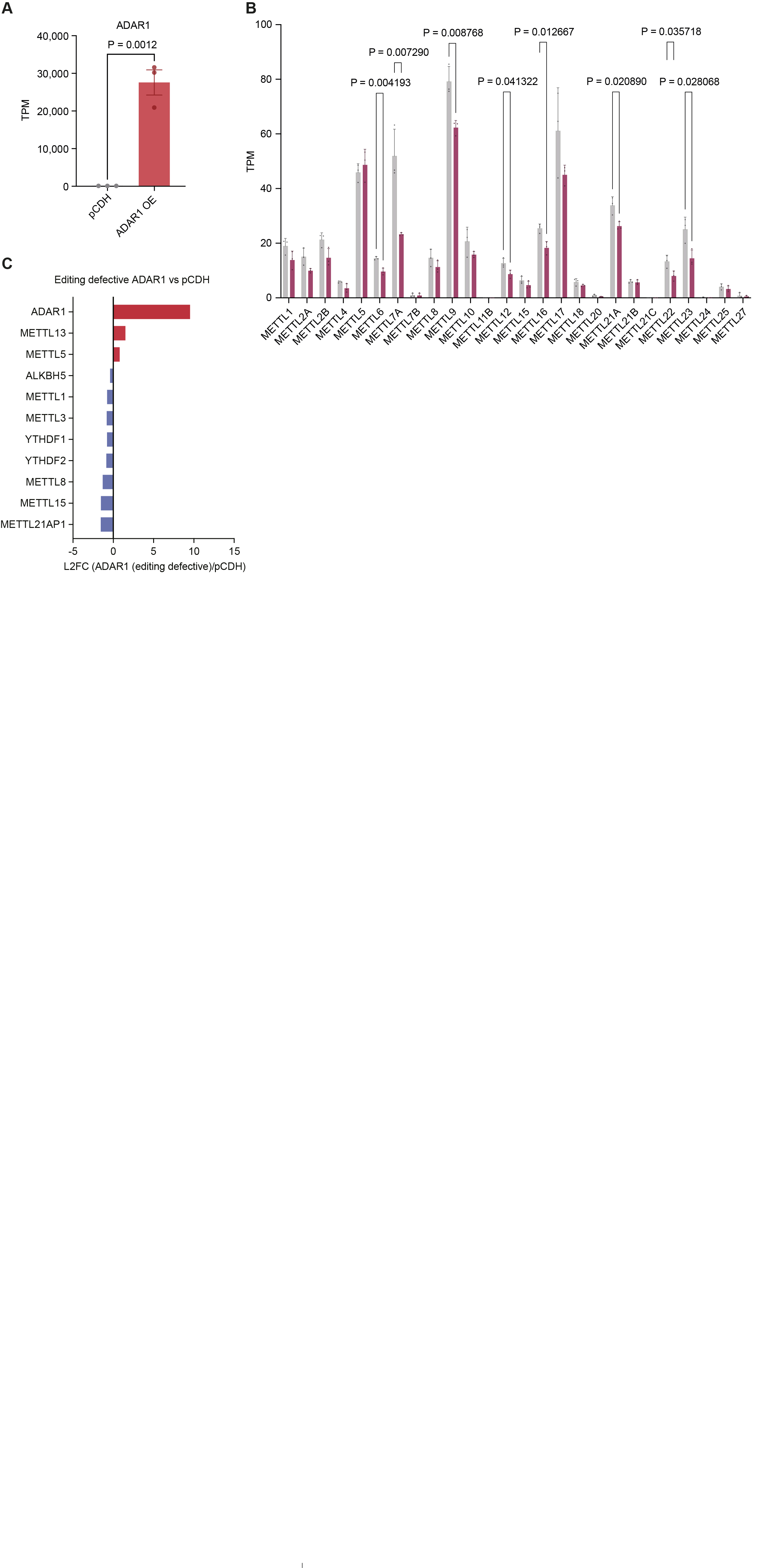

Supplement: Supplementary file 2 — Supplemental Figure 1 [file 41419_2026_8761_MOESM2_ESM.png]

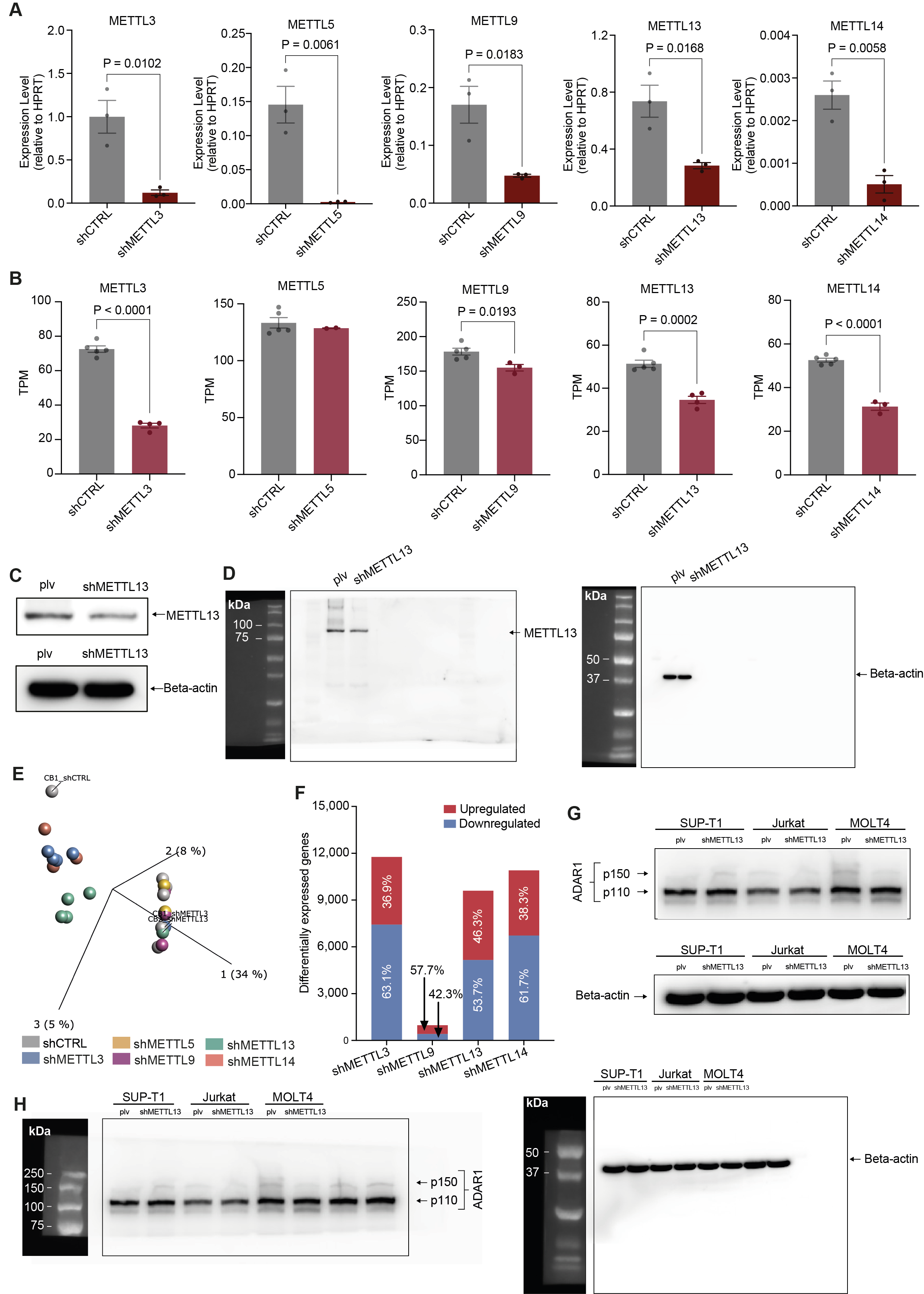

Supplement: Supplementary file 3 — Supplemental Figure 2 [file 41419_2026_8761_MOESM3_ESM.png]

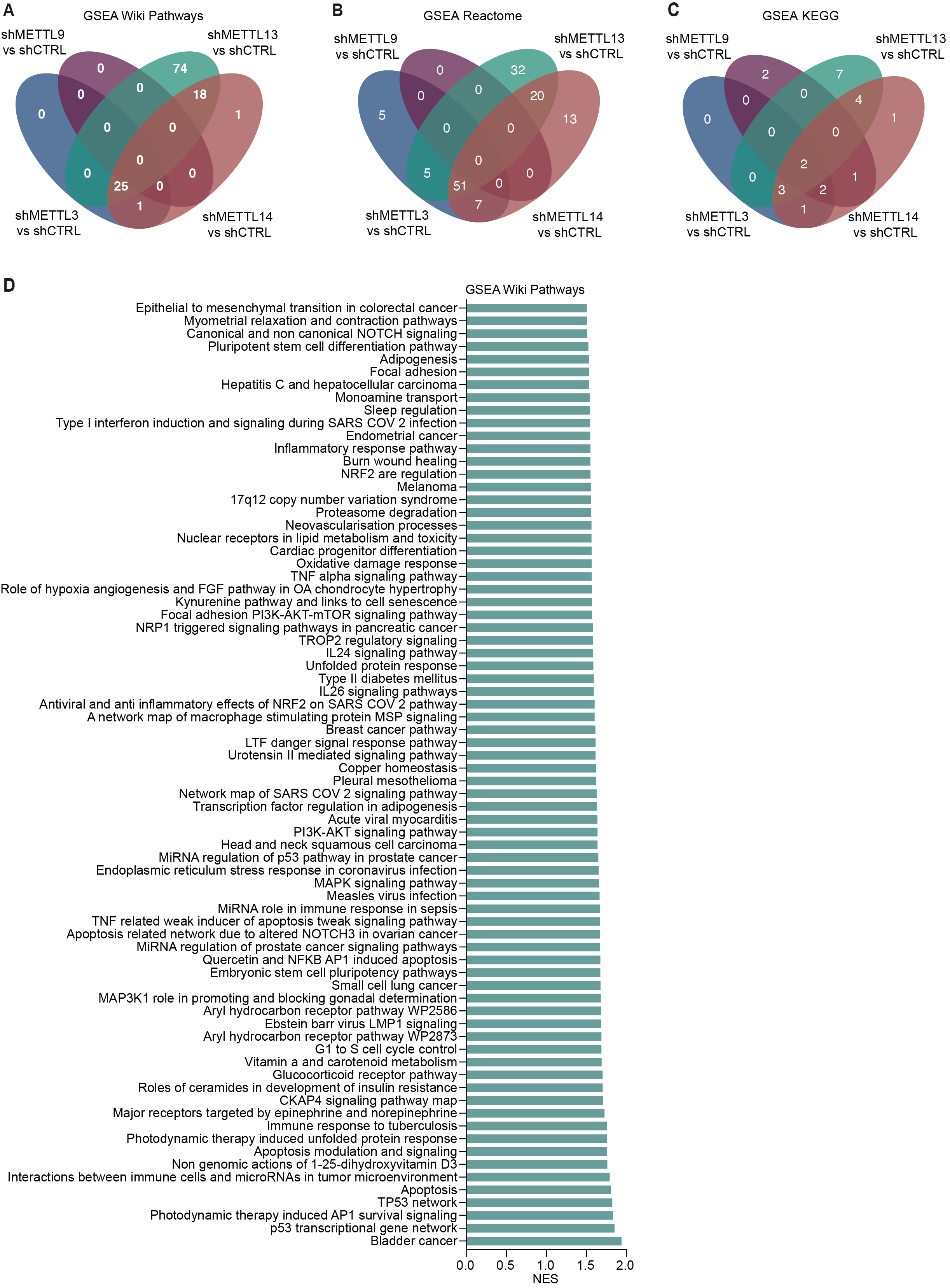

Supplement: Supplementary file 4 — Supplemental Figure 3 [file 41419_2026_8761_MOESM4_ESM.png]

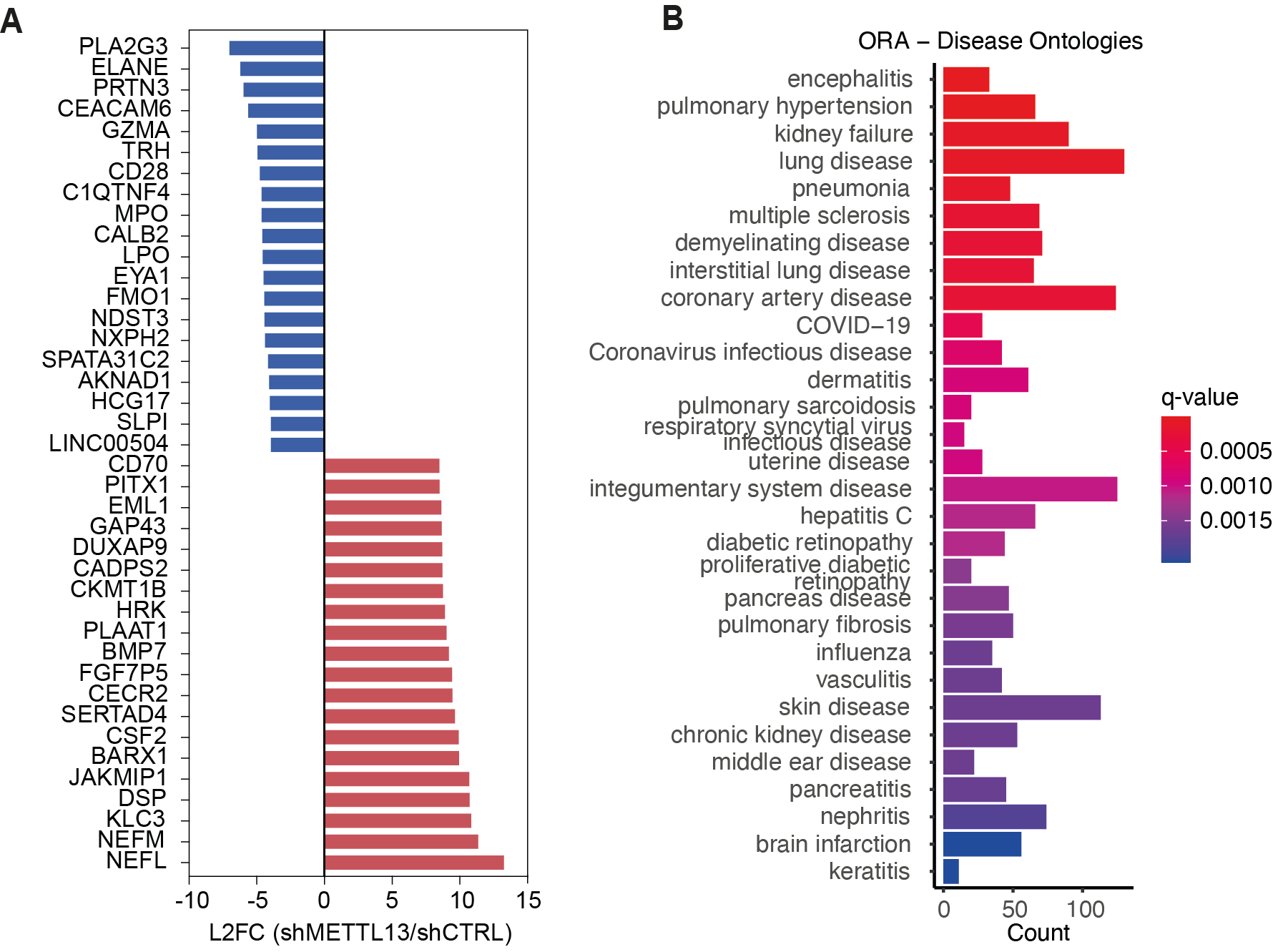

Supplement: Supplementary file 5 — Supplemental Figure 4 [file 41419_2026_8761_MOESM5_ESM.png]

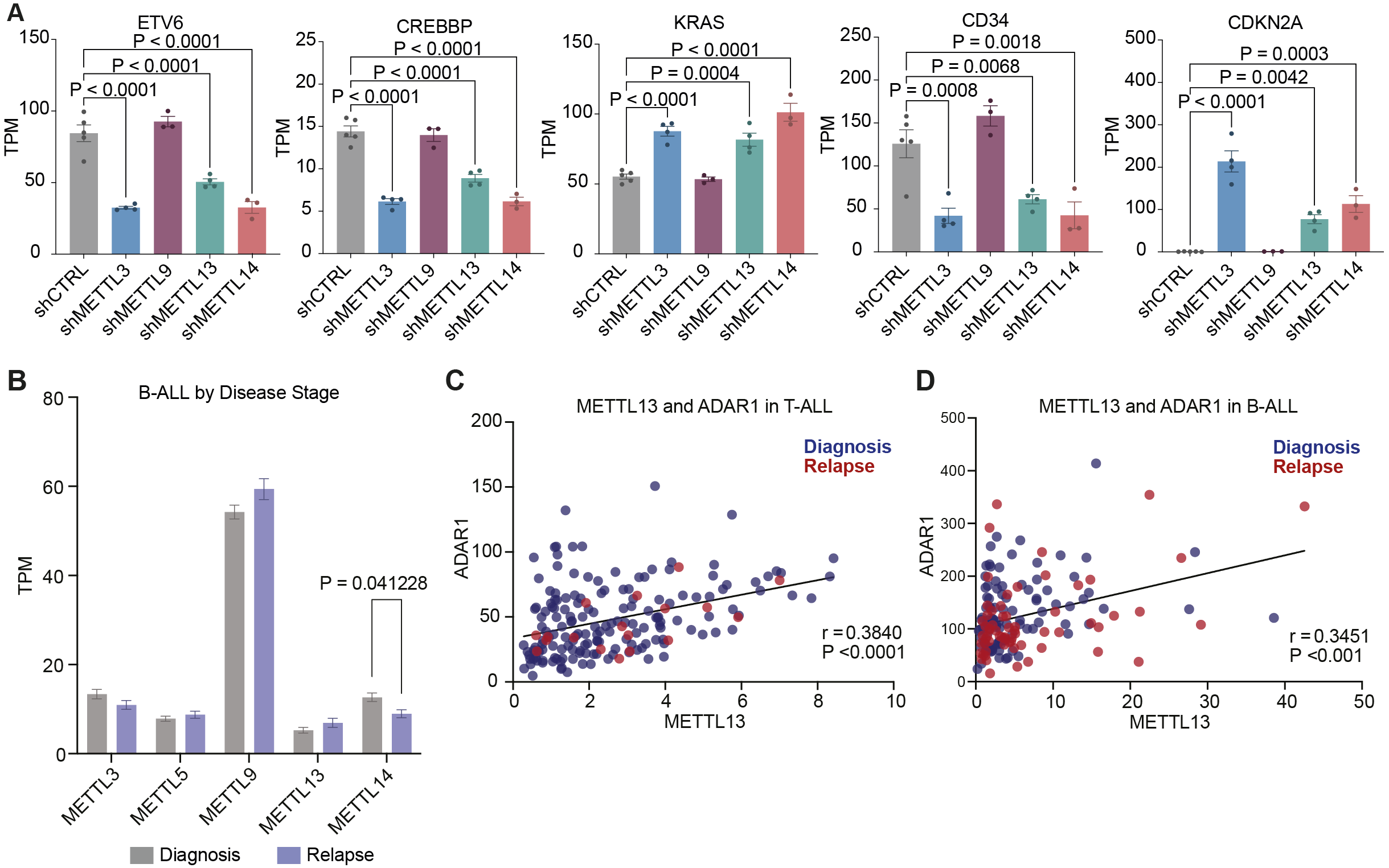

Supplement: Supplementary file 6 — Supplemental Figure 5 [file 41419_2026_8761_MOESM6_ESM.png]

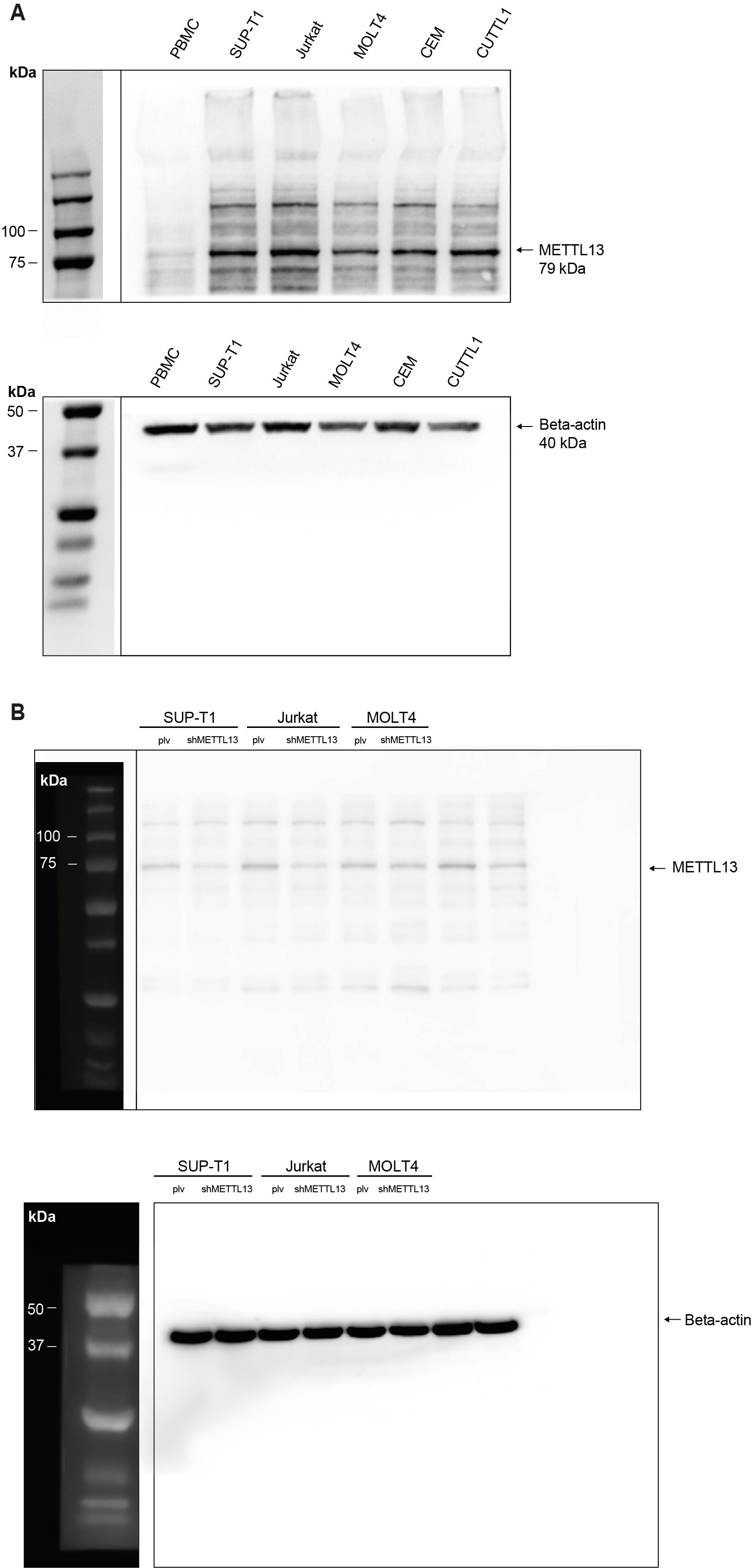

Supplement: Supplementary file 7 — Supplemental Figure 6 [file 41419_2026_8761_MOESM7_ESM.png]

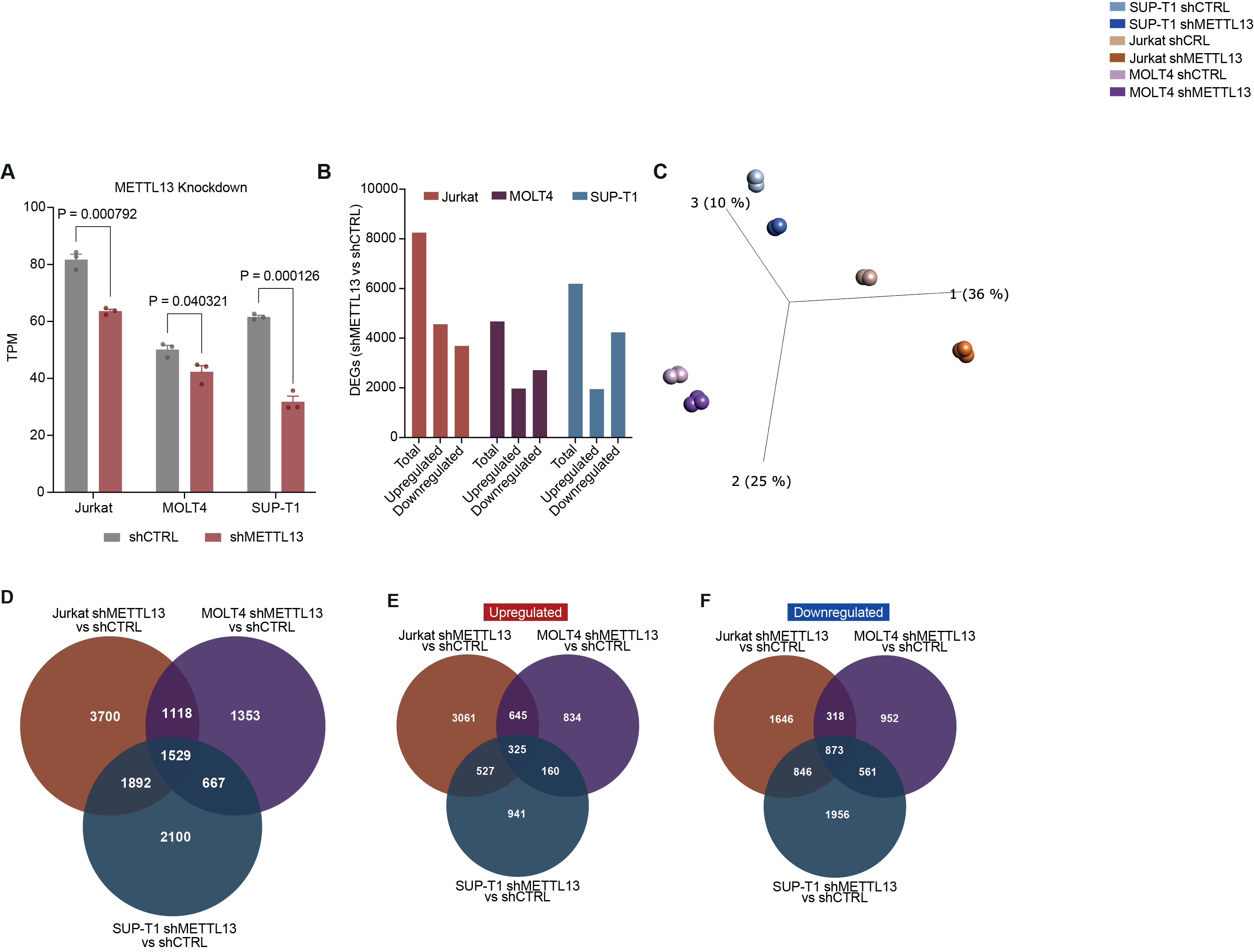

Supplement: Supplementary file 8 — Supplemental Figure 7 [file 41419_2026_8761_MOESM8_ESM.png]

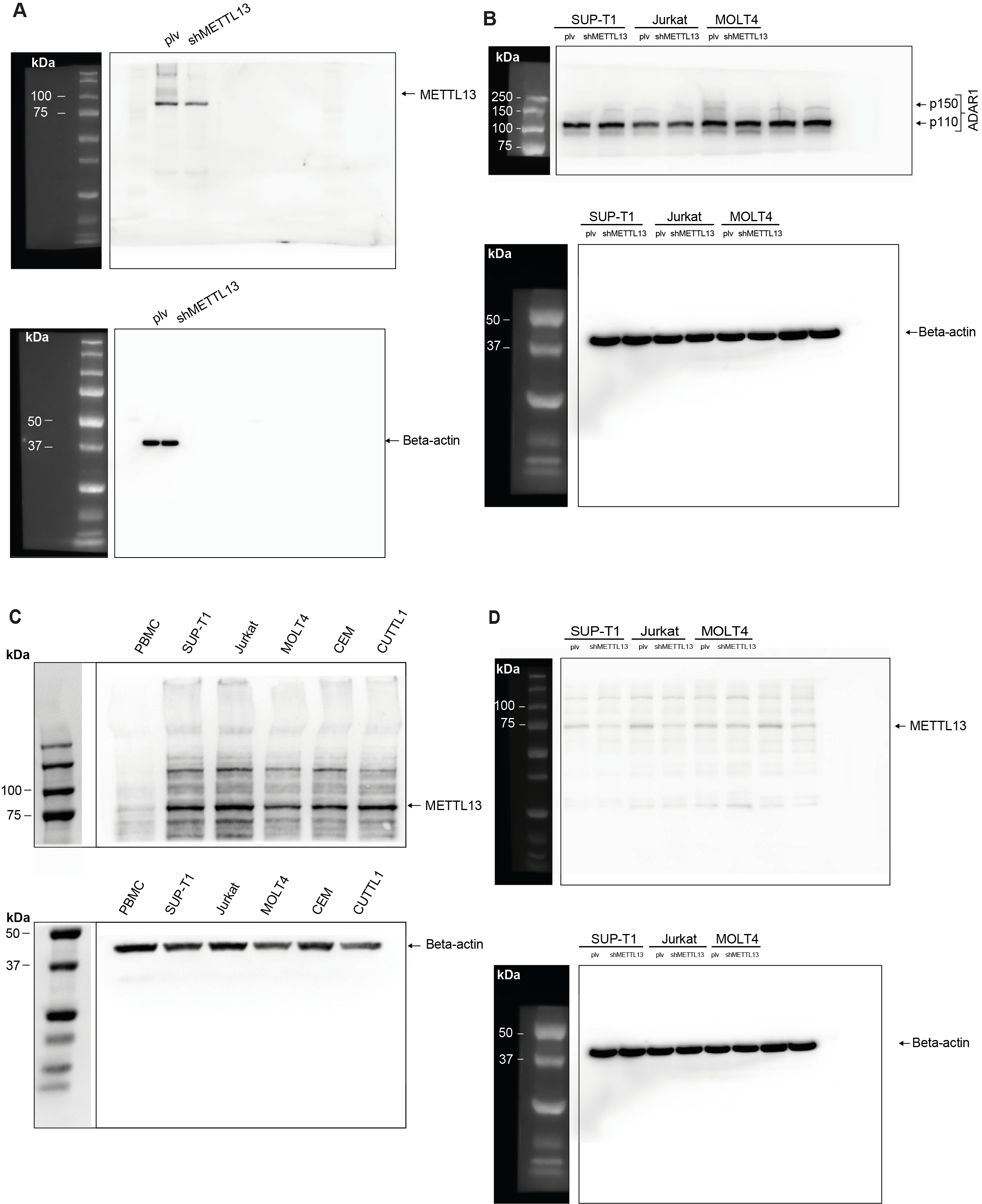

Supplement: Supplementary file 14 — Uncropped western blots [file 41419_2026_8761_MOESM14_ESM.png]
